# Supplementary material for: Mammalian cells measure the extracellular matrix area and respond through switching the adhesion state
Source: Nat Commun. 2025 Jul 25;16:6870. doi: 10.1038/s41467-025-62153-7 (PMC12297719; doi:10.1038/s41467-025-62153-7)
Supplement: Supplementary file 1 — Supplementary Information [file 41467_2025_62153_MOESM1_ESM.docx]

**Supplementary Information**


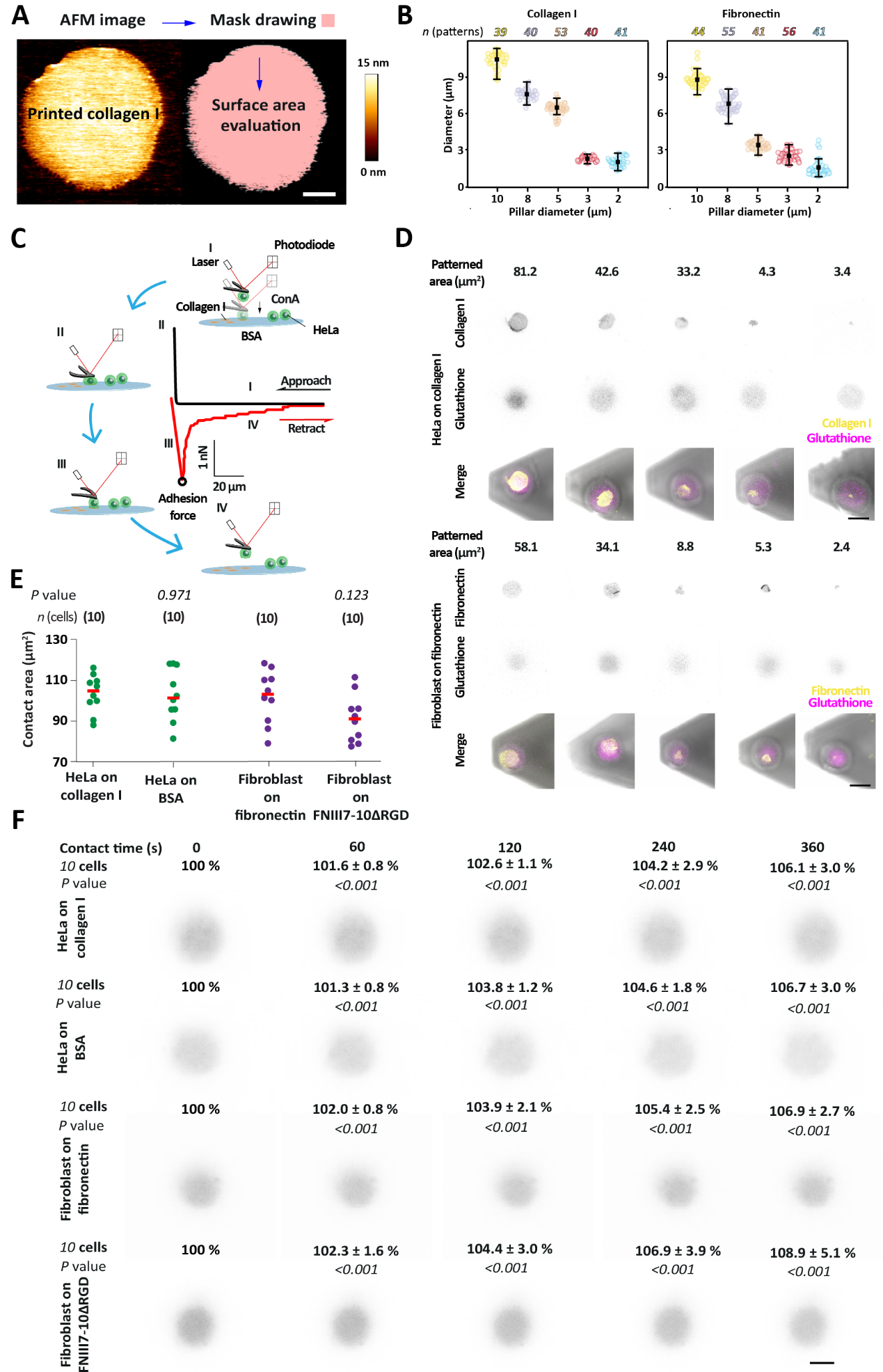


**Supplementary Fig. 1. AFM-based SCFS setup to measure ECM protein area dependent cell adhesion forces.** (A) Evaluation of surface area from AFM topographs of a collagen I pattern at the half average height of the collagen I protruding from the glass support. Scale bar, 2 μm. (B) Average diameter of collagen I or fibronectin patterns printed using PDMS pillars having the diameters indicated. Data points represents the average diameters (*d*_1_, *d*_2_; see Fig. 2) of one pattern. Black dots represent averages from *n* patterns and error bars standard deviation. (C) For Single-cell force spectroscopy (SCFS), a single, rounded cell is a attached to a ConA-coated microcantilever (Methods) (I). The microcantilever-attached cell is approached to an ECM protein pattern until reaching ≈ 2 nN contact force, where it is kept for a given contact time (II). The cantilever is retracted (III) to detach the cell from the patterned substrate (IV). During approach and retraction, the deflection of the cantilever is recorded to record force-distance (FD) curves. The maximum downward deflection in the retraction FD curve quantifies the cell adhesion force. (D) Confocal microscopy images of a microcantilever-attached (top) HeLa cell or (bottom) fibroblast. Cells were fluorescently labelled using CellTracker (pink) and printed collagen I or fibronectin patterns were labeled using Alexa-fluor-555 (yellow). *n* = 5 independent experiments for each condition. Scale bars, 10 μm. (E) Contact area of HeLa cells (green) and fibroblasts (purple) to indicated substrate at the initial contact with the substrate. Dots represent contact areas of single cells, red bars median values, and *n* (cells) the number of individual cells measured. *P* values were calculated by two sided Mann-Whitney tests and compare the contact area of HeLa cells to collagen I and BSA or of fibroblasts to fibronectin and FNIII7-10ΔRGD. (F) Representative timelapse confocal microscopy images of CellTracker labeled single rounded HeLa cells or fibroblasts attached to a ConA-coated cantilever in contact with the given substrate for the indicated contact time (*n* (cells)=10). Contact area changes (mean ± SD) are indicated in percent for each contact time. *P* values were calculated by two-sided paired *t*-tests and compare the contact area at the given contact time with the contact area at 0 s contact time. Scale bar, 10 μm.


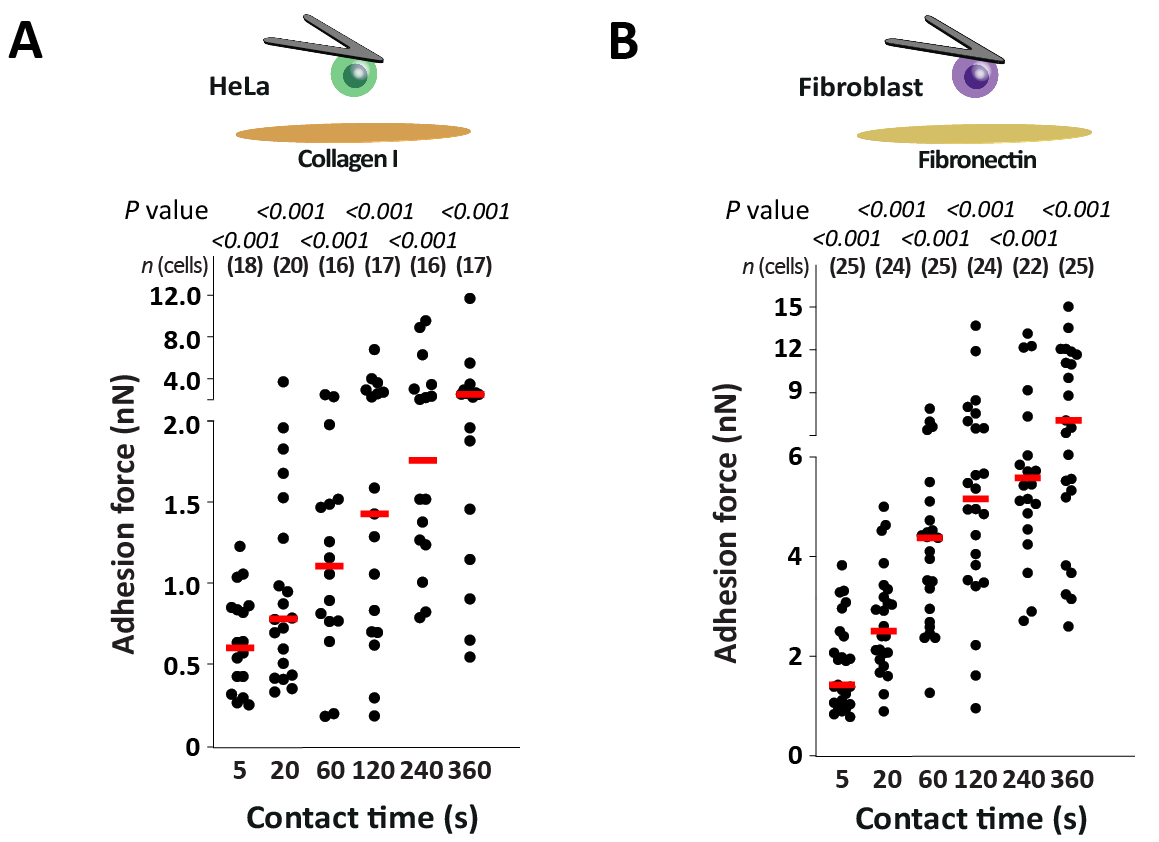


**Supplementary Fig. 2. Adhesion force of HeLa cells and fibroblasts to unrestricted ECM substrates.** (A,B) Adhesion force of (A) wild type (wt) HeLa cells to unrestricted collagen I substrates and (B) wt fibroblasts to unrestricted fibronectin substrates at given contact times. Dots represent adhesion forces of single cells, red bars median values, and *n* (cells) the number of individual cells tested in at least three independent experiments. *P* values were calculated using two-sided Mann-Whitney tests and compare adhesion force of displayed data with adhesion force of wt HeLa cells to unrestricted BSA substrates or of wt fibroblasts to FNIII7-10ΔRGD substrates.


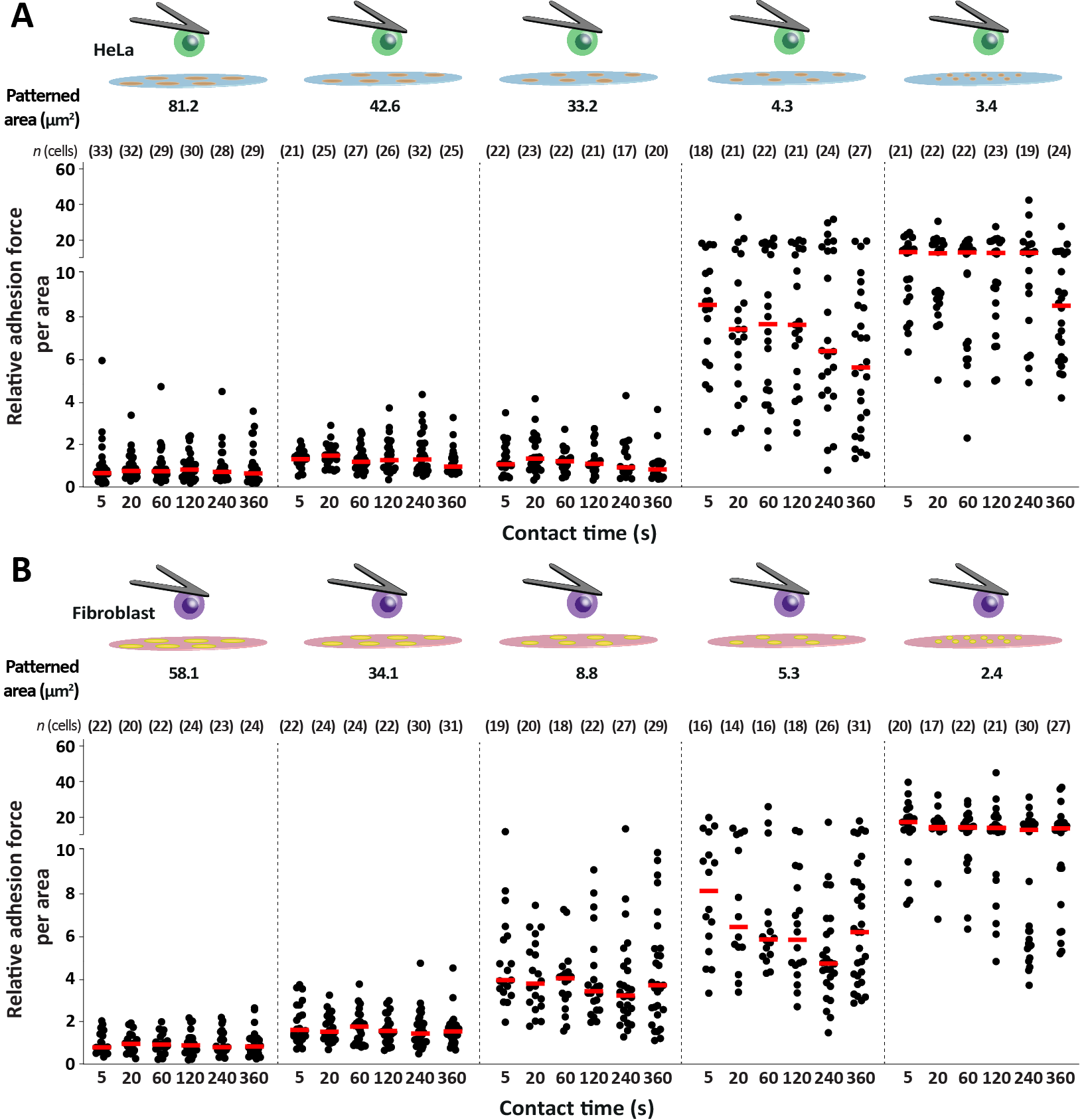


**Supplementary Fig. 3. The cell adhesion force per substrate area increases with decreasing area of the printed ECM protein pattern.** (A,B) Relative adhesion force of (A) wt HeLa cells or (B) wt fibroblasts per area of printed (A) collagen I or (B) fibronectin patterns. The adhesion forces were normalized to the mean cell adhesion force per area on the largest substrate pattern and contact time (data taken from Fig. 4). Dots represent relative adhesion forces per area of single cells, red bars median values, and *n* (cells) the number of individual cells measured in at least three independent experiments. Statistical analysis comparing the adhesion force per area at 5 s contact time with longer contact times is given in Supplementary Table 7 and 8.


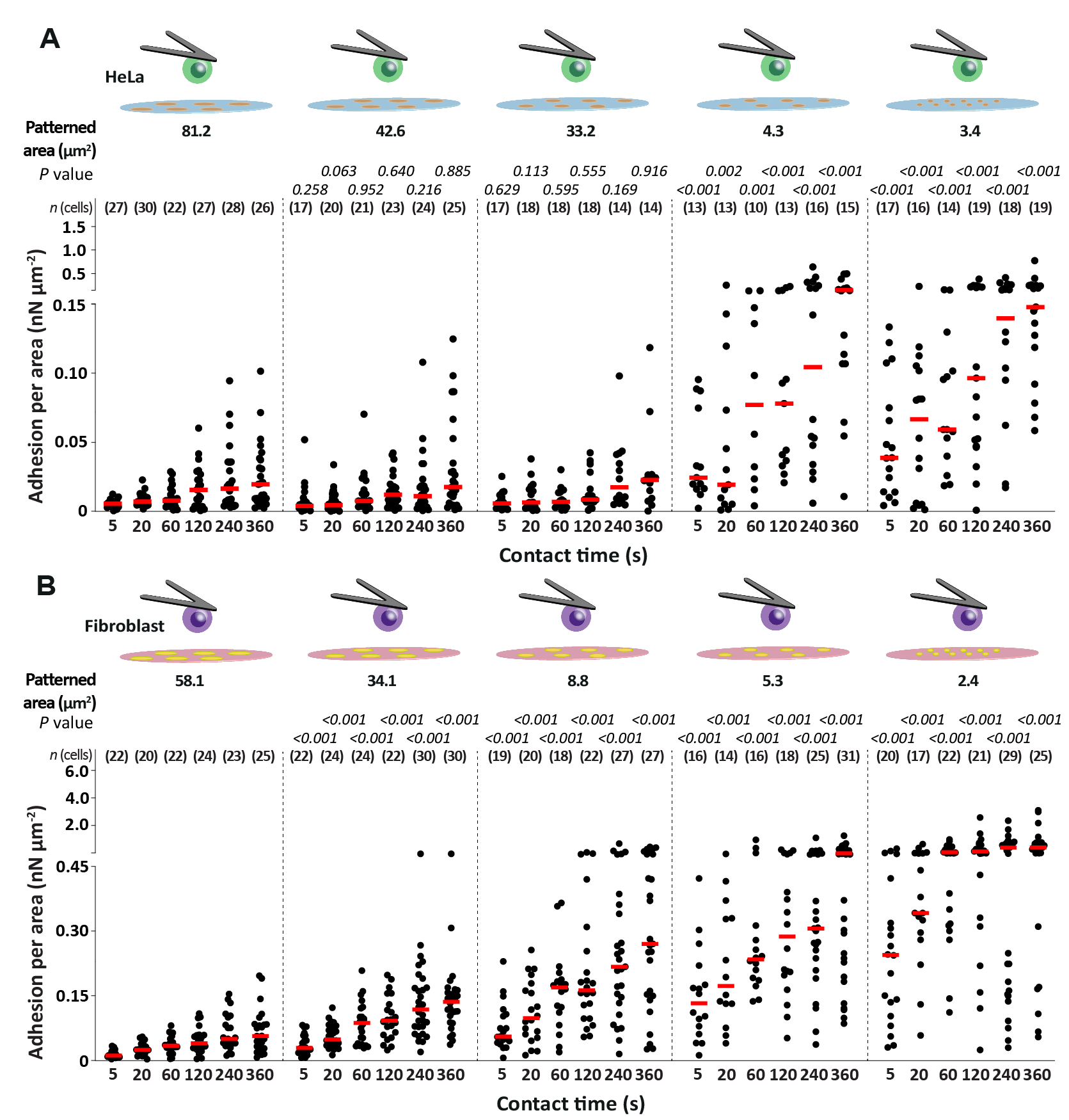
**Supplementary Fig. 4. Even after correction for possible unspecific adhesion contribution, the cell adhesion force per area increases with decreasing area of the printed ECM protein pattern.** To estimate the integrin-specific adhesion force of cells to patterned ECM protein substrates, the mean adhesion force at the given contact time of (A) wt HeLa cells to BSA or (B) wt fibroblasts to FNIII7-10ΔRGD was subtracted from the adhesion force of individual cells to the ECM protein patterns for given contact times (data taken from Fig. 3). Subsequently the corrected adhesion forces were normalized to the area of the ECM protein pattern (data taken from Fig. 2). Dots represent adhesion forces of single cells per area of ECM protein pattern, red bars median values, and *n* (cells) the number of independent cells tested in at least three independent experiments. *P* values were calculated by two-sided Mann-Whitney tests and compare the displayed data and the adhesion force per area of (A) wt HeLa cells to 81.2 μm^2^ large collagen I patterns or (B) wt fibroblasts to 58.1 μm^2^ large fibronectin patterns.


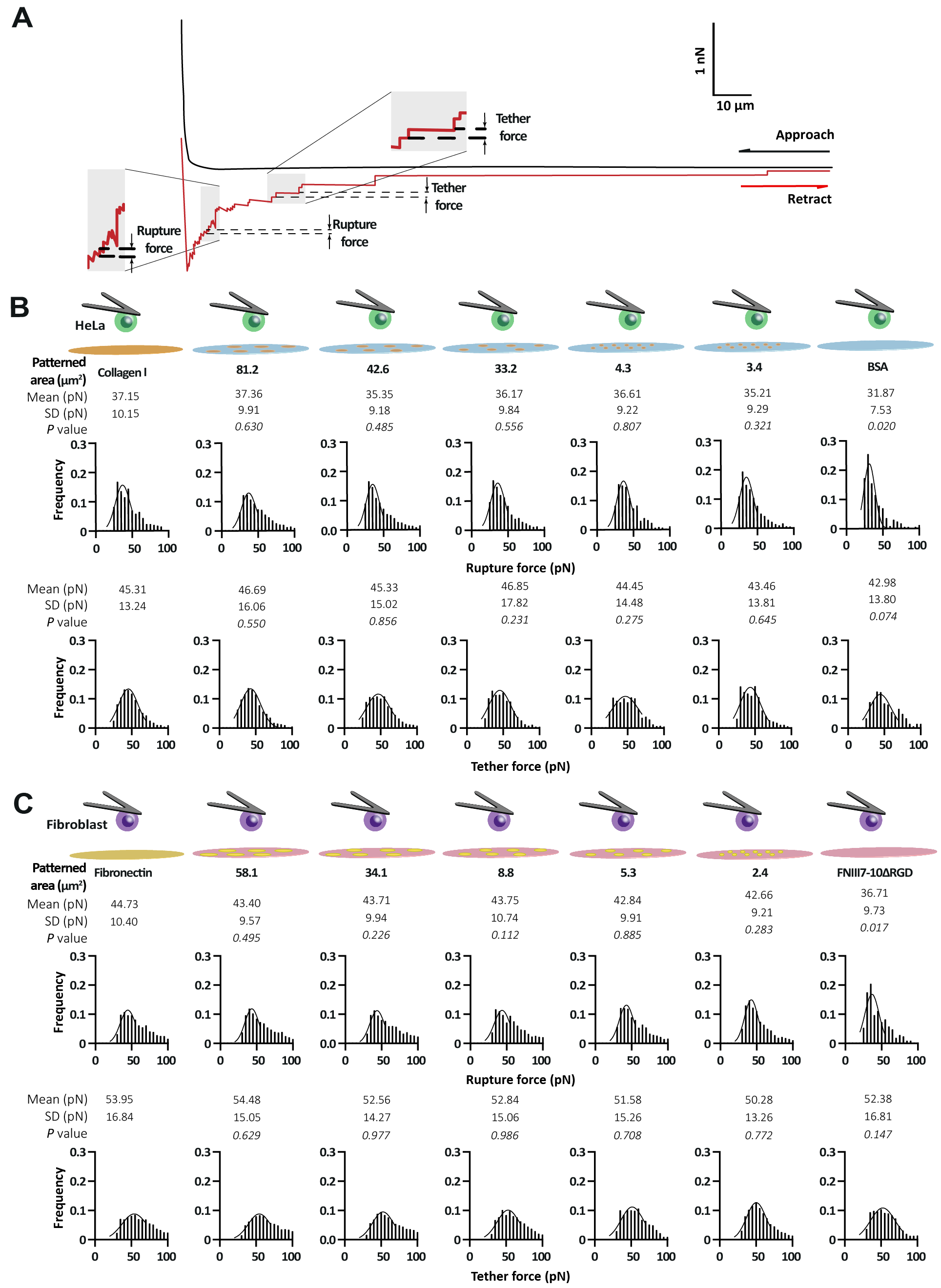


**Supplementary Fig. 5. Rupture force and tether force are independent of the area of the ECM protein pattern.** (A) FD curves recorded upon detaching wt HeLa cells from the substrate display multiple smaller unbinding events after the adhesion force peak. These unbinding events correspond to single rupture and tether events (2x magnified in grey background). A rupture event quantifies the unbinding force of a single or multiple adhesion receptors from the ligand. A tether event represents the extrusion of a membrane tether from the cell until the receptor-ligand bond that anchors the tether to the ECM substrate ruptures^1^. To analyze rupture and tether events, the force-distance curves recorded were pooled across all contact times for each pattern size indicated. (B, C) Rupture force (top row) and tether force (bottom row) distributions of (B) wt HeLa cells adhering to collagen I patterns or of (C) wt fibroblasts adhering to fibronectin patterns with indicated area. Lines depict Gaussian functions used to fit the unbinding force distributions. *P* values were calculated using the one-sided extra sum-of-squares *F* test and compare the peak values of displayed Gaussian fits to those derived from unrestricted (B) collagen I or (C) fibronectin.


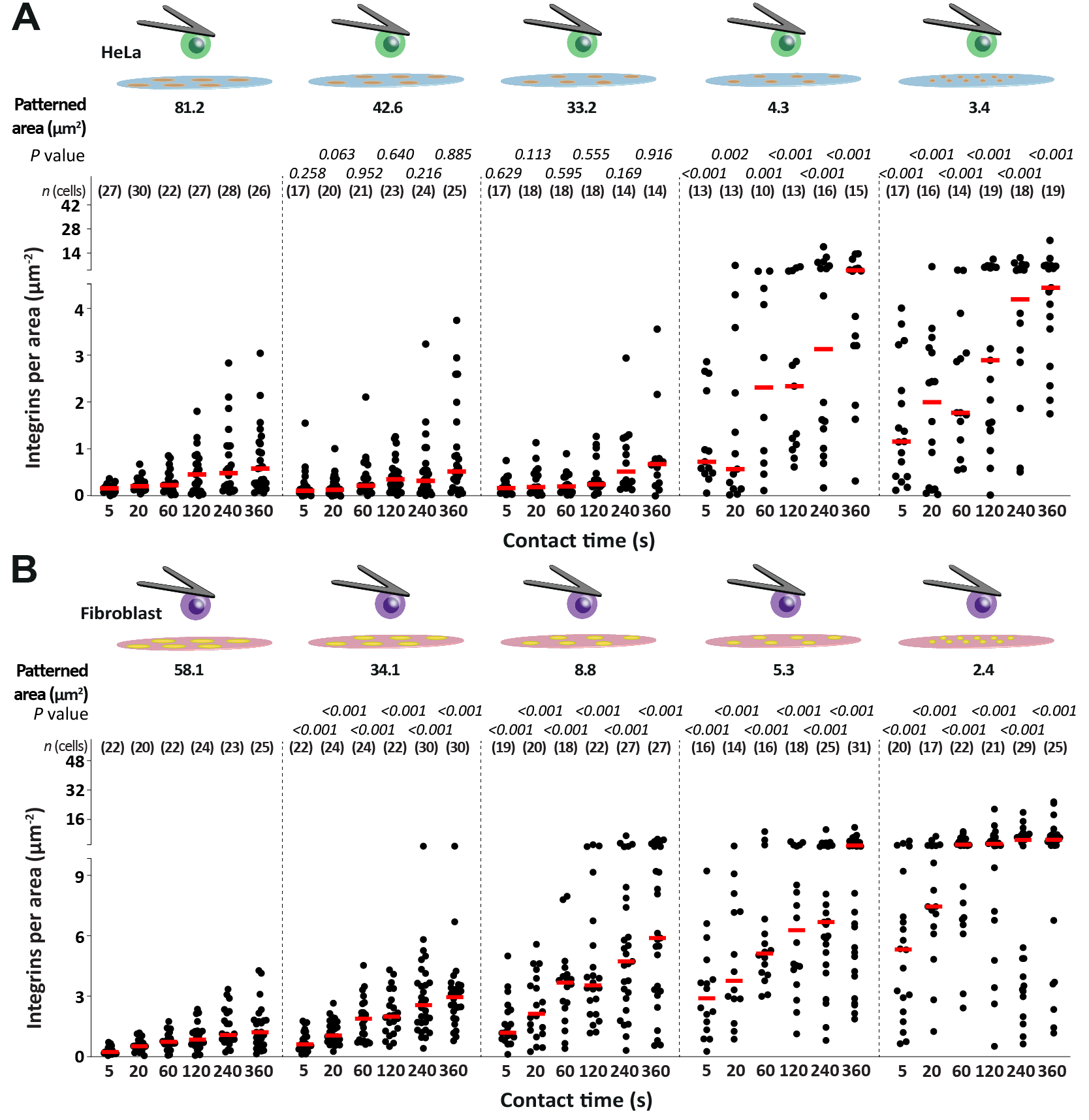


**Supplementary Fig. 6. Density of ligand-bound integrins increases with decreasing substrate area.** (A, B) Ligand-bound integrins per area (density) for (A) wt HeLa cells adhering to collagen I patterns and (B) wt fibroblasts adhering to fibronectin patterns. Ligand-bound integrin densities were estimated by dividing the cell adhesion force per patterned substrate area (data taken from Supplementary Fig. 4A,B) by the average unbinding force of single integrins adhering to unrestricted (A) collagen I or (B) fibronectin substrates. Dots represent densities of ligand-bound integrins of single cells, red bars median values, and *n* (cells) the number of individual cells tested in at least three independent experiments. Ligand-bound integrins per area for (A) wt HeLa cells adhering to 81.2 μm^2^ large collagen I patterns or (B) wt fibroblasts adhering to 58.1 μm^2^ large fibronectin patterns are given as reference (semitransparent). *P* values were calculated using two-sided Mann-Whitney tests and compare the ligand-bound integrin density of displayed data with reference.


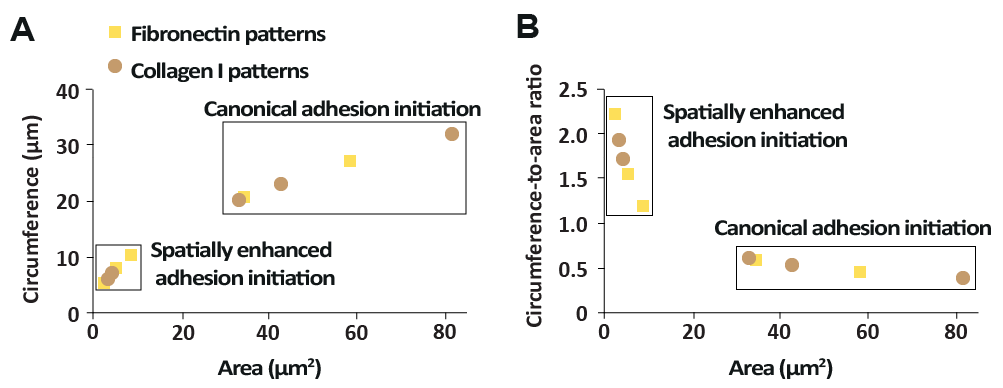


**Supplementary Fig. 7. The circumference of the ECM protein pattern affects how cells initiate adhesion.** (A) Circumference and (B) circumference-to-area ratio of ECM protein patterns having different areas. Data points present circumferences calculated from average areas of ECM protein patterns (data taken from Fig. 2D). The boxes indicate to which ECM protein patterns cells initiate adhesion canonically or spatially enhanced.


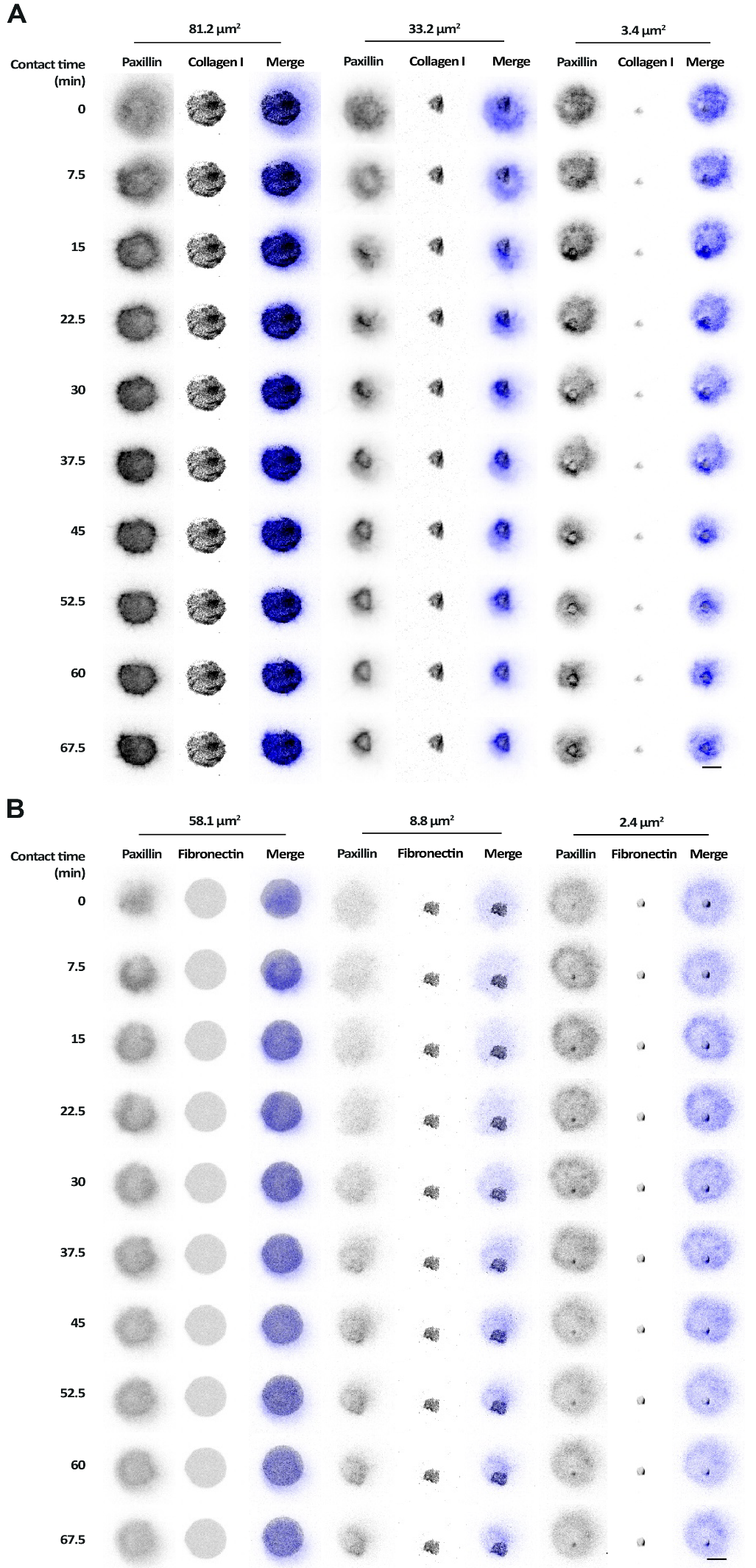


**Supplementary Fig. 8. The formation of adhesion sites by HeLa cells on collagen I patterns and by fibroblasts on fibronectin patterns depends on the pattern size.** (A,B) Representative timelapse confocal microscopy images of paxillin-GFP expressing (A) HeLa cells on collagen I patterns and (B) of fibroblasts on fibronectin patterns (*n* = 5 independent experiments for each condition). Pattern size (area) and contact time are indicated. A single, rounded paxillin-GFP expressing (A) HeLa cell or (B) fibroblast was attached to a ConA-coated microcantilever and then brought into contact with collagen I or fibronectin patterns until reaching contact times of ≈ 70 min. Collagen I or fibronectin patterns were labelled using Alexa fluor 555 (gray). Scale bars, 5 μm.


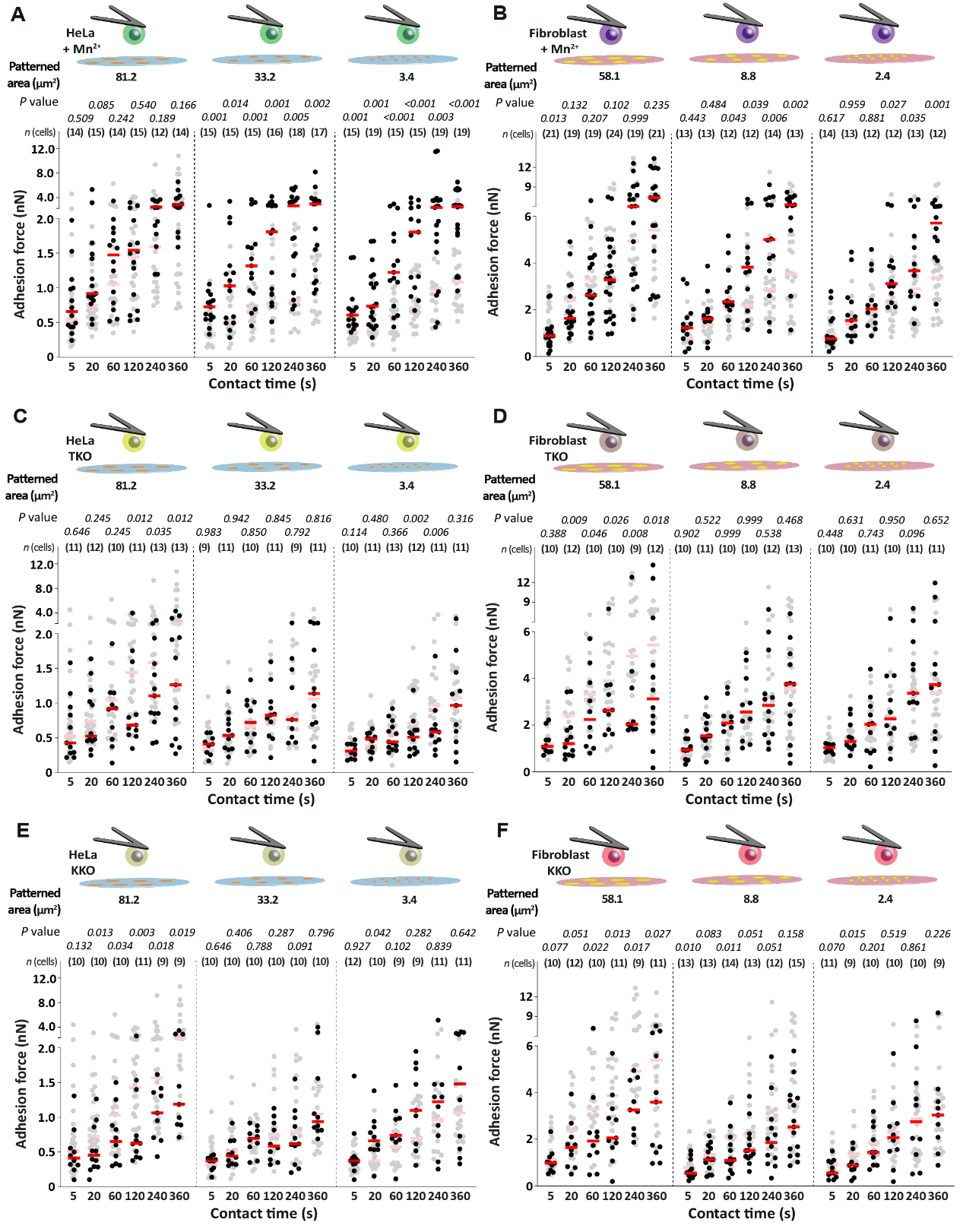


**Supplementary Fig. 9. HeLa cells and fibroblasts require integrin activation to sense and respond to area of the ECM pattern.** (A-F) Adhesion force of (A) Mn^2+^-treated wt HeLa cells, (B) Mn^2+^-treated wt fibroblasts, (C) talin1/2-depleted (TKO) HeLa cells, (D) TKO fibroblasts, (E) kindlin1/2-depleted (KKO) HeLa cells, and (F) KKO fibroblasts to different areas of collagen I or fibronectin patterns at given contact times. Dots represent adhesion forces per area of individual cells, red bars the median, and *n* (cells) the number of individual cells tested in at least three independent experiments. Adhesion force of untreated wt HeLa cells or wt fibroblasts in the respective condition is given as reference (semitransparent). *P* values were calculated by two-sided Mann-Whitney tests and compare the adhesion forces of given data with the reference data.


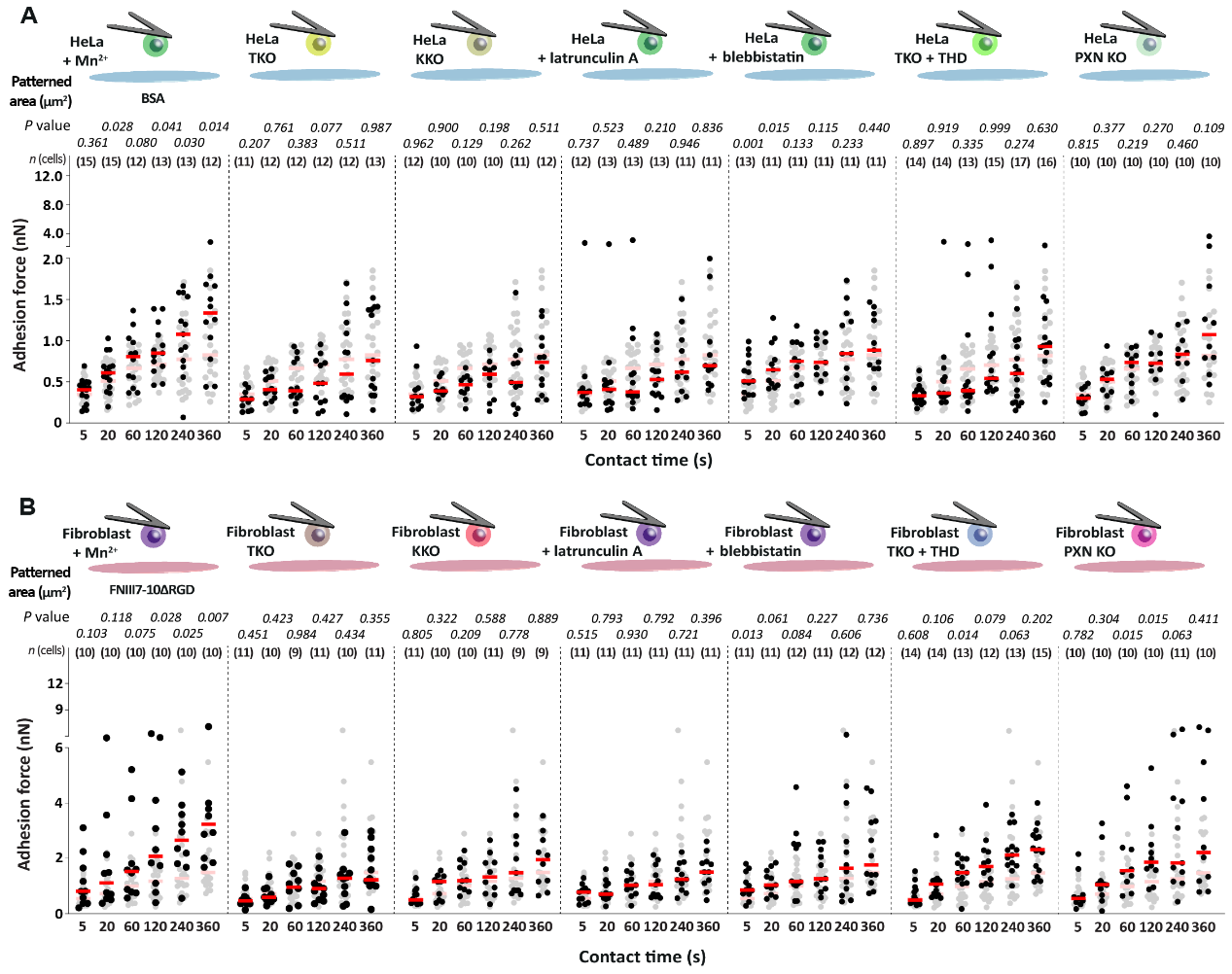


**Supplementary Fig. 10. Engineered HeLa cells and fibroblasts establish similar unspecific adhesion to unrestricted BSA and FNIII7-10ΔRGD** **substrates.** (A, B) Adhesion force of (A) Mn^2+^-treated, talin1/2-depleted (TKO), kindlin1/2-depleted (KKO), latrunculin A-treated wt, blebbistatin-treated wt, TKO expressing talin1 head domain (TKO+THD), and paxillin-depleted (PXN KO) HeLa cells to BSA or of (B) Mn^2+^-treated, TKO, KKO, latrunculin A-treated wt, blebbistatin-treated wt, TKO+THD, and PXN KO fibroblasts to FNIII7-10ΔRGD substrates at given contact times. Dots represent adhesion force of single cells, red bars median values, and *n* (cells) the number of individual cells tested in at least three independent experiments. The adhesion force of (A) wt HeLa cells to unrestricted BSA or (B) wt fibroblasts to unrestricted FNIII7-10ΔRGD substrates is given as reference (semitransparent). *P* values were calculated using two-sided Mann-Whitney tests and compare the adhesion force of displayed with reference data.


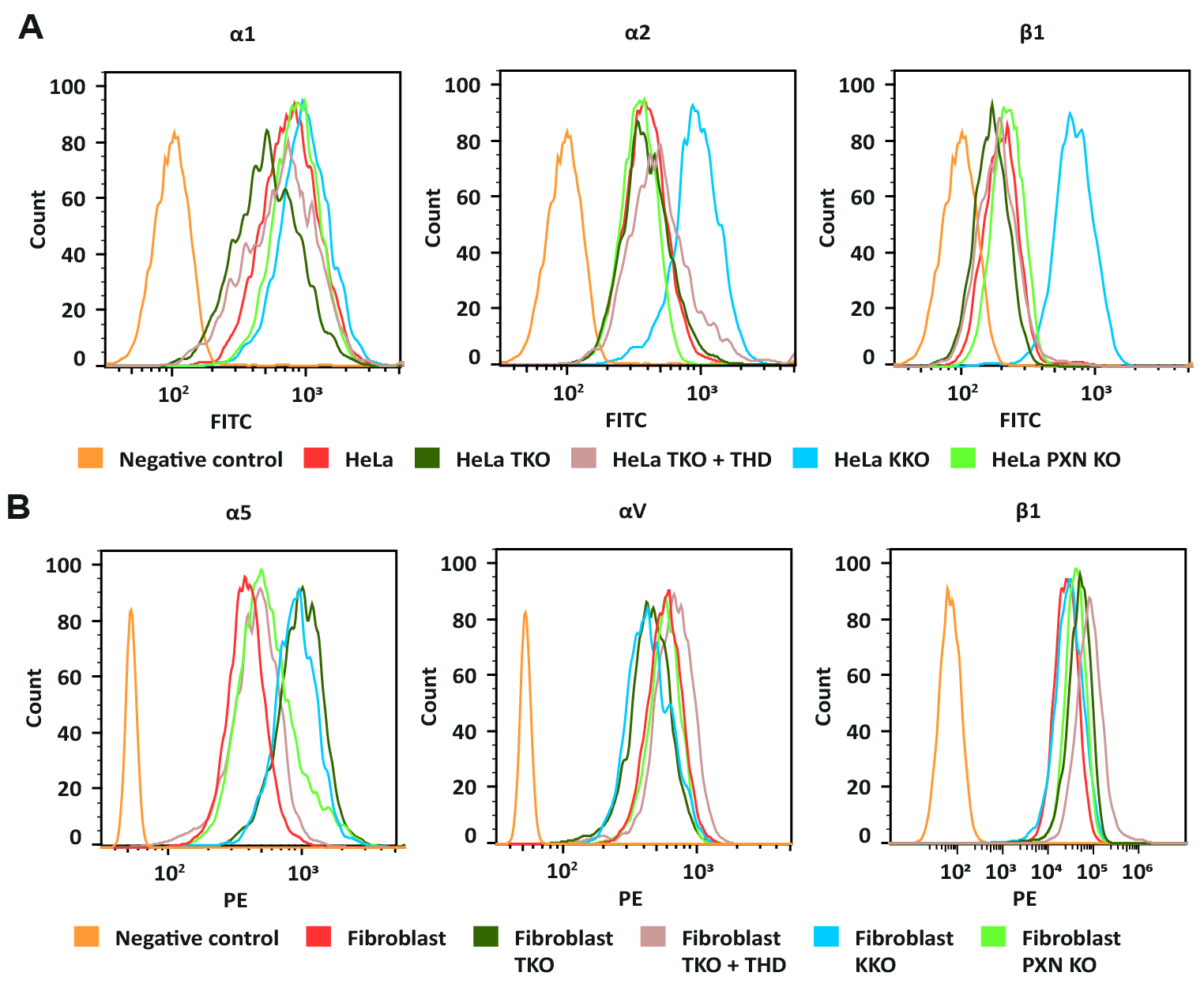


**Supplementary Fig. 11. Integrin surface expression does not majorly affect the results of SCFS experiments.** (A,B) Integrin surface expression level of integrin subunits characterized by flow cytometry. Fluorescence intensity of (A) wt HeLa cells, talin1/2-depleted (TKO) HeLa cells, kindlin1/2-depleted (KKO) HeLa cells, TKO expressing talin1 head domain (TKO+THD) HeLa cells, and paxillin-depleted (PXN KO) HeLa cells, and of (B) wt fibroblasts, TKO fibroblasts, KKO fibroblasts, TKO +THD fibroblasts, and PXN KO fibroblasts. Unstained (A) wt HeLa cells and (B) wt fibroblasts were used as negative controls. *n* = 3 independent experiments for each condition.


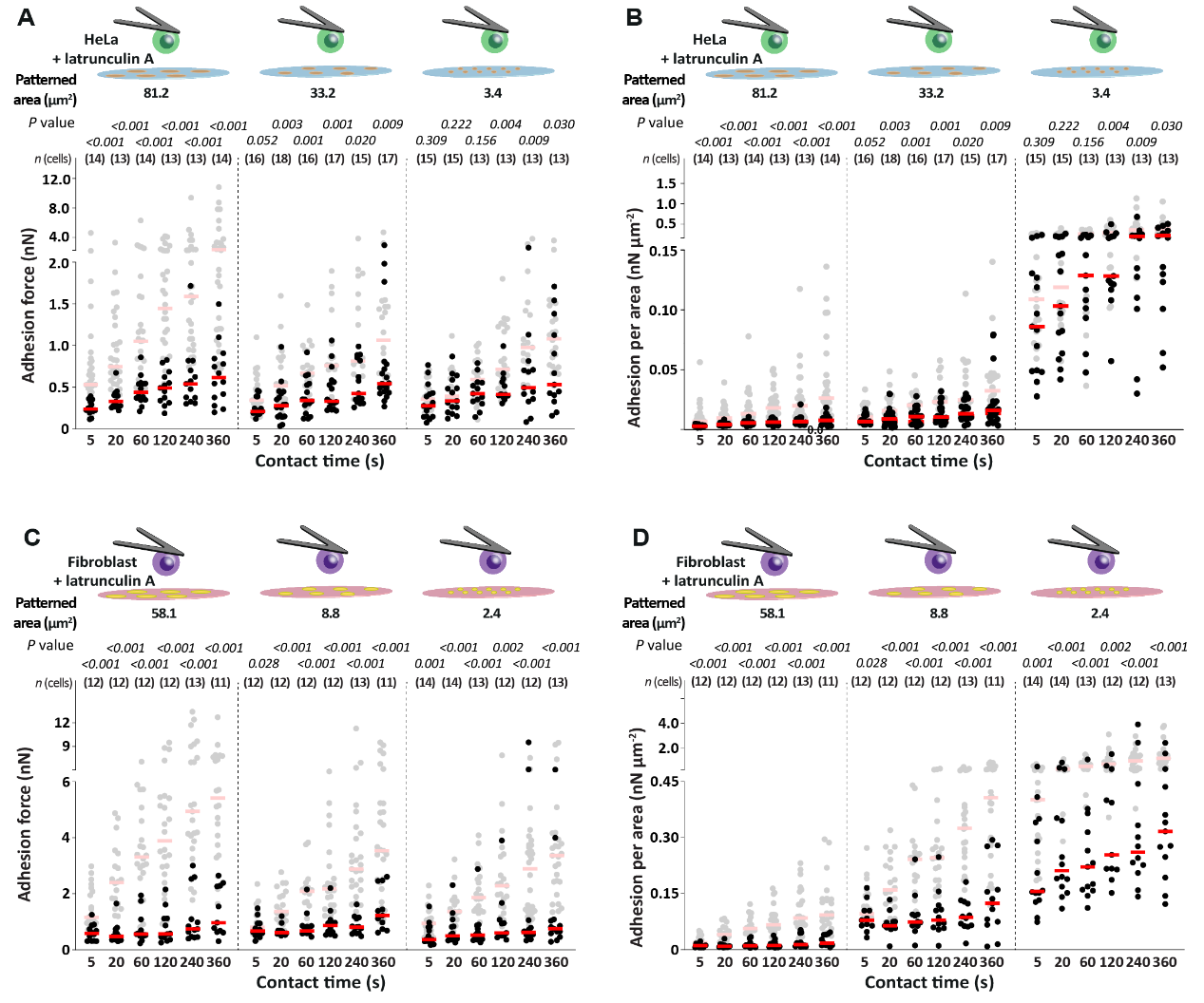


**Supplementary Fig. 12. The spatially enhanced adhesion state requires an intact actin cytoskeleton.** (A-D) Adhesion force and adhesion force per area of 1 µM latrunculin A-treated (A,B) wt HeLa cells and (C,D) wt fibroblasts to different areas of collagen I or fibronectin patterns at given contact times. Dots represent adhesion forces of individual cells, red bars the median, and *n* (cells) the number of individual cells tested in at least three independent experiments. (A,C) Adhesion force and (B,D) adhesion force per area of untreated wt HeLa cells or wt fibroblasts are given as reference (semitransparent). *P* values were calculated by two-sided Mann-Whitney tests and compare the given adhesion forces with the reference data.


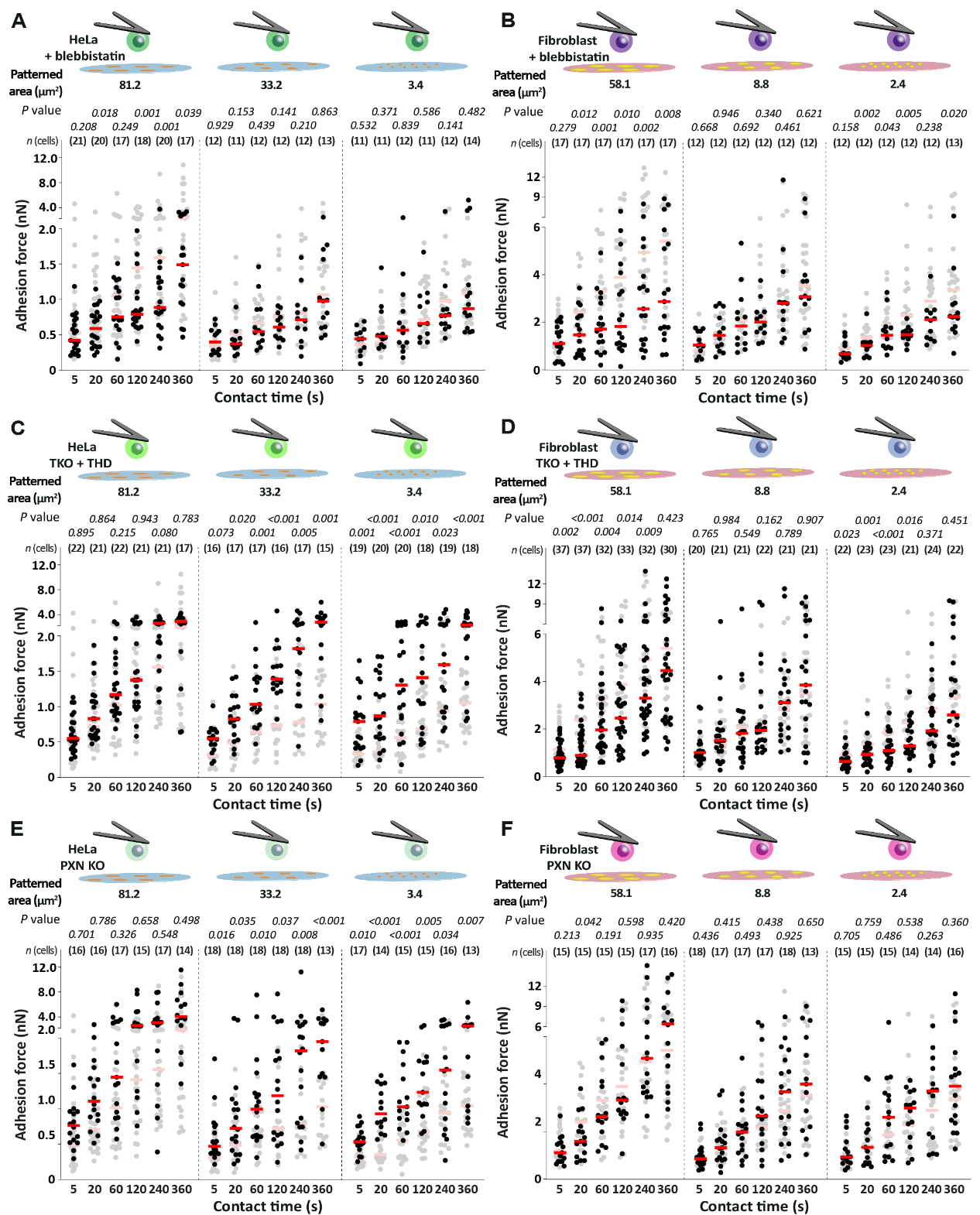


**Supplementary Fig. 13. Actin-cytoskeleton and adhesome formation plays specific and different role in HeLa cells and fibroblasts.** (A-F) Adhesion force of (A) 20 µM blebbistatin-treated HeLa cells, (B) 20 µM blebbistatin-treated fibroblasts, (C) talin1/2-depleted expressing talin1 head domain (TKO+THD) HeLa cells, (D) TKO+THD fibroblasts, (E) paxillin-depleted (PXN KO) HeLa cells, and (F) PXN KO fibroblasts to different areas of collagen I or fibronectin patterns at given contact times. Dots represent adhesion force per area of individual cells, red bars the median, and *n* (cells) the number of individual cells tested in at least three independent experiments. Adhesion force of un-treated wt HeLa cells or wt fibroblasts in the respective condition is given as reference (semitrans-parent). *P* values were calculated by two-sided Mann-Whitney tests and compare the adhesion forces of given data with the reference data. Statistical analysis of the adhesion force established by TKO+THD and PXN KO HeLa cells to collagen patterns or TKO+THD and PXN KO fibroblasts to fibronectin patterns is given in Supplementary Table 11,12.


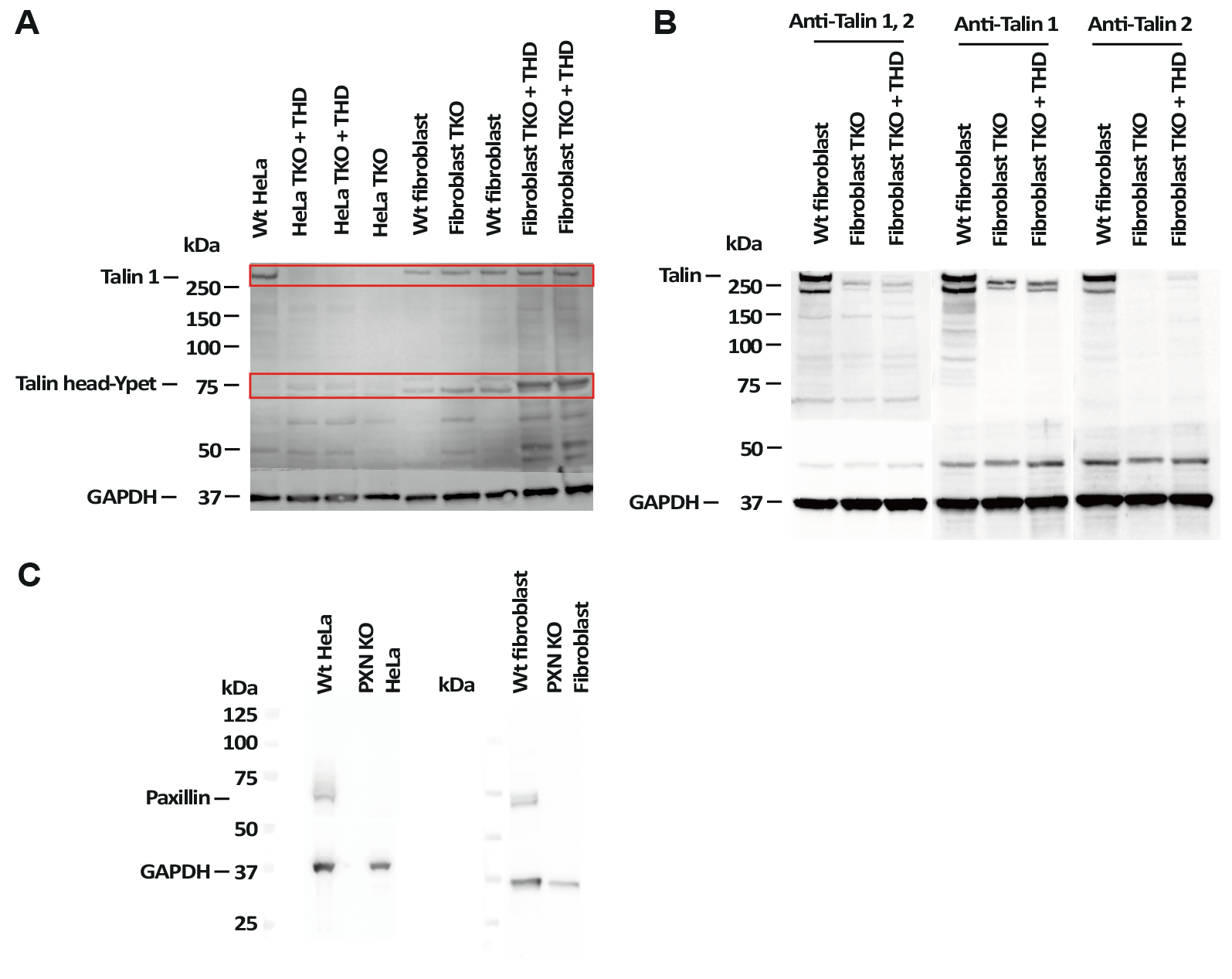


**Supplementary Fig. 14. Verification of talin1 head expression and paxillin depletion in HeLa and fibroblast cell lines.** (A) Western blot of cell lysates of wt, talin1/2-depleted (TKO), TKO expressing talin1 head domain (TKO+THD) HeLa cells and wt, TKO, TKO+THD fibroblasts using anti-talin [TA205]. The specific band of full length talin (≈ 270 kDa) is detected in wt HeLa cells. The specific band of human talin head-Ypet (≈ 75 kDa) is detected in TKO+THD HeLa cells and TKO+THD fibroblasts. The band (≈ 300 kDa) is presented in wt, TKO, TKO+THD fibroblasts. Because the anti-talin [TA205] does not react with mouse species, this band is considered as unspecific. *n* = 3 independent experiments). (B) Western blot of cell lysates of wt, TKO, and TKO+THD fibroblasts using anti-talin1and 2 [8D4], anti-talin1 [97H6], and anti-talin2 [68E7] antibodies. The specific band of full length talin (≈ 270 kDa) is only detected in wt fibroblasts. A weak band at ≈ 230 kDa is detected of TKO fibroblasts and TKO + THD fibroblasts, are derived from different sources (Methods). Because this band does not differentiate between both cell lines, it is considered unspecific. *n* = 3 independent experiments. (C) Western blot of cell lysates of wt HeLa cells, paxillin KO (PXN KO) HeLa cells, wt fibroblasts, and PXN KO fibroblasts using anti-paxillin and GAPDH antibodies. While a specific band of ≈ 68 kDa is detected in wt HeLa cells and wt fibroblasts cell by the anti-paxillin antibody, it is not detectable in the knockout cell lines. *n* = 3 independent experiments. All samples (A-C) were using anti-GAPDH antibodies as a loading control.


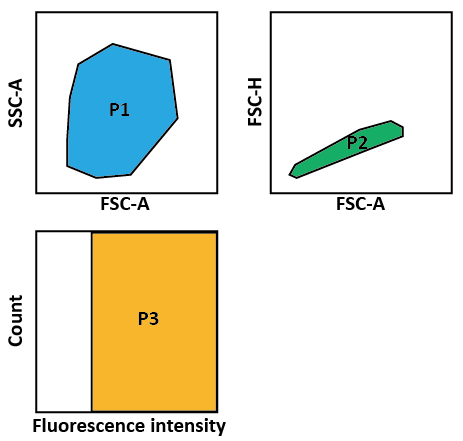


**Supplementary Fig. 15. Gating strategy for flow cytometry experiments.** Debris was excluded according to side scatter area (SSC-A) versus forward scatter area FSC-A and the depicted population P1 was analyzed further. Doublets were excluded by forward scatter height (FSC-H) versus FSC-A and depicted population P2 was further analyzed. The depicted population P3 was used to analyze the median fluorescence intensity of the cell population.

| Analysis of printed collagen I patterns | | | |
| --- | --- | --- | --- |
| Pillar diameter | *n* | Mean | Standard deviation |
| Height (nm) | | | |
| 2 | 41 | 14.2 | 5.0 |
| 3 | 40 | 10.4 | 2.6 |
| 5 | 53 | 6.2 | 2.1 |
| 8 | 40 | 9.8 | 1.2 |
| 10 | 39 | 9.7 | 2.3 |
| Diameter (μm) | | | |
| 2 | 41 | 2.1 | 0.4 |
| 3 | 40 | 2.4 | 0.2 |
| 5 | 53 | 6.5 | 0.5 |
| 8 | 40 | 7.6 | 0.6 |
| 10 | 39 | 10.5 | 0.7 |
| Roundness | | | |
| 2 | 41 | 0.93 | 0.17 |
| 3 | 40 | 1.01 | 0.10 |
| 5 | 53 | 1.01 | 0.06 |
| 8 | 40 | 0.98 | 0.06 |
| 10 | 39 | 1.01 | 0.07 |
| Area (μm^2^) | | | |
| 2 | 41 | 3.4 | 1.1 |
| 3 | 40 | 4.3 | 0.8 |
| 5 | 53 | 33.2 | 3.6 |
| 8 | 40 | 42.6 | 6.2 |
| 10 | 39 | 81.2 | 9.2 |

**Supplementary Table 1. Analysis of the height, diameter, roundness and areas of printed collagen I patterns.** Height, diameter, roundness, and surface area of printed collagen I patterns. Values give mean and standard deviation. *n* gives the number of printed collagen I patterns analyzed.

| Analysis of printed fibronectin patterns | | | |
| --- | --- | --- | --- |
| Pillar diameter | *n* | Mean | Standard deviation |
| Height (nm) | | | |
| 2 | 41 | 7.3 | 2.0 |
| 3 | 56 | 6.0 | 2.0 |
| 5 | 41 | 4.9 | 0.6 |
| 8 | 55 | 5.1 | 0.8 |
| 10 | 44 | 6.5 | 1.0 |
| Diameter (μm) | | | |
| 2 | 41 | 1.6 | 0.7 |
| 3 | 56 | 2.6 | 0.4 |
| 5 | 41 | 3.5 | 0.4 |
| 8 | 55 | 6.7 | 0.6 |
| 10 | 44 | 8.8 | 0.6 |
| Roundness | | | |
| 2 | 41 | 1.01 | 0.14 |
| 3 | 56 | 1.03 | 0.10 |
| 5 | 41 | 0.99 | 0.09 |
| 8 | 55 | 1.01 | 0.08 |
| 10 | 44 | 0.98 | 0.06 |
| Area (μm^2^) | | | |
| 2 | 41 | 2.4 | 2.2 |
| 3 | 56 | 5.3 | 1.4 |
| 5 | 41 | 8.8 | 1.9 |
| 8 | 55 | 34.1 | 4.6 |
| 10 | 44 | 58.1 | 7.7 |

**Supplementary Table 2. Analysis of the height, diameter, roundness and area of printed fibronectin patterns.** Height, diameter, roundness, and area of printed fibronectin patterns. Values give mean and standard deviation. *n* gives the number of printed fibronectin patterns analyzed.

| Comparison of the adhesion force of wt HeLa cells to printed collagen I patterns | | | | | | | |
| --- | --- | --- | --- | --- | --- | --- | --- |
| Patterned area (μm^2^) | | 81.2 | | | | | |
| Contact time (s) | | 5 | 20 | 60 | 120 | 240 | 360 |
| Median cell adhesion force (nN) | | 0.540 | 0.756 | 1.060 | 1.455 | 1.600 | 2.120 |
| Standard error (nN) | | 0.140 | 0.102 | 0.220 | 0.183 | 0.344 | 0.512 |
| Patterned area 42.6 μm^2^ | *P* value | 0.235 | 0.207 | 0.166 | 0.107 | 0.399 | 0.264 |
| Patterned area 33.2 μm^2^ | *P* value | 0.017 | 0.006 | 0.008 | <0.001 | 0.005 | <0.001 |
| Patterned area 4.3 μm^2^ | *P* value | 0.004 | <0.001 | 0.002 | <0.001 | <0.001 | <0.001 |
| Patterned area 3.4 μm^2^ | *P* value | 0.008 | <0.001 | <0.001 | <0.001 | 0.005 | <0.001 |
| Patterned area (μm^2^) |  | 42.6 | | | | | |
| Contact time (s) | | 5 | 20 | 60 | 120 | 240 | 360 |
| Median cell adhesion force (nN) | | 0.519 | 0.715 | 0.819 | 1.095 | 1.415 | 1.690 |
| Standard error (nN) | | 0.044 | 0.051 | 0.075 | 0.134 | 0.181 | 0.208 |
| Patterned area 33.2 μm^2^ | *P* value | 0.187 | 0.094 | 0.119 | 0.020 | 0.016 | 0.008 |
| Patterned area 4.3 μm^2^ | *P* value | 0.049 | <0.001 | 0.029 | 0.010 | 0.005 | <0.001 |
| Patterned area 3.4 μm^2^ | *P* value | 0.109 | <0.001 | 0.004 | 0.013 | 0.034 | <0.001 |
| Patterned area (μm^2^) |  | 33.2 | | | | | |
| Contact time (s) | | 5 | 20 | 60 | 120 | 240 | 360 |
| Median cell adhesion force (nN) | | 0.342 | 0.518 | 0.668 | 0.759 | 0.814 | 1.067 |
| Standard error (nN) | | 0.052 | 0.074 | 0.062 | 0.098 | 0.207 | 0.221 |
| Patterned area 4.3 μm^2^ | *P* value | 0.677 | 0.057 | 0.325 | 0.584 | 0.560 | 0.302 |
| Patterned area 3.4 μm^2^ | *P* value | 0.919 | 0.302 | 0.246 | 0.839 | 0.661 | 0.731 |
| Patterned area (μm^2^) |  | 4.3 | | | | | |
| Contact time (s) | | 5 | 20 | 60 | 120 | 240 | 360 |
| Median cell adhesion force (nN) | | 0.344 | 0.364 | 0.535 | 0.671 | 0.708 | 0.904 |
| Standard error (nN) | | 0.040 | 0.072 | 0.084 | 0.099 | 0.185 | 0.145 |
| Patterned area 3.4 μm^2^ | *P* value | 0.530 | 0.334 | 0.991 | 0.733 | 0.326 | 0.254 |
| Patterned area (μm^2^) |  | 3.4 | | | | | |
| Contact time (s) | | 5 | 20 | 60 | 120 | 240 | 360 |
| Median cell adhesion force (nN) | | 0.367 | 0.401 | 0.601 | 0.723 | 0.983 | 1.085 |
| Standard error (nN) | | 0.034 | 0.046 | 0.054 | 0.081 | 0.186 | 0.119 |

**Supplementary Table 3. Statistical analysis comparing the adhesion force of wt HeLa cells to printed collagen I patterns with indicated substrate area at given contact time.** Given are the median and standard error of the adhesion forces of wt HeLa cells to printed collagen I patterns for different contact times. *P* values comparing adhesion forces of wt HeLa cells to printed collagen patterns of given area with that measured of the indicated collagen I pattern area at the given contact time. *P* values were calculated using two-sided Mann-Whitney tests, black values show significant difference (*P* < 0.05), and red values non-significant difference (*P* ≥ 0.05). Data taken from Fig. 3A.

| Comparison of the adhesion force of wt fibroblasts to printed fibronectin patterns | | | | | | | |
| --- | --- | --- | --- | --- | --- | --- | --- |
| Patterned area (μm^2^) | | 58.1 | | | | | |
| Contact time (s) | | 5 | 20 | 60 | 120 | 240 | 360 |
| Median cell adhesion force (nN) | | 1.180 | 2.445 | 3.350 | 3.930 | 4.980 | 5.455 |
| Standard error (nN) | | 0.154 | 0.267 | 0.345 | 0.481 | 0.707 | 0.844 |
| Patterned area 34.1 μm^2^ | *P* value | 0.379 | 0.939 | 0.935 | 0.944 | 0.615 | 0.916 |
| Patterned area 8.8 μm^2^ | *P* value | 0.034 | 0.003 | <0.001 | 0.004 | <0.001 | 0.022 |
| Patterned area 5.3 μm^2^ | *P* value | 0.268 | 0.050 | 0.008 | 0.009 | <0.001 | 0.009 |
| Patterned area 2.4 μm^2^ | *P* value | 0.022 | 0.006 | <0.001 | 0.003 | <0.001 | 0.002 |
| Patterned area (μm^2^) |  | 34.1 | | | | | |
| Contact time (s) | | 5 | 20 | 60 | 120 | 240 | 360 |
| Median cell adhesion force (nN) | | 1.370 | 2.235 | 3.645 | 3.960 | 5.070 | 5.790 |
| Standard error (nN) | | 0.171 | 0.193 | 0.336 | 0.369 | 0.563 | 0.495 |
| Patterned area 8.8 μm^2^ | *P* value | 0.011 | <0.001 | 0.002 | 0.002 | <0.001 | 0.003 |
| Patterned area 5.3 μm^2^ | *P* value | 0.077 | 0.015 | 0.007 | 0.005 | <0.001 | <0.001 |
| Patterned area 2.4 μm^2^ | *P* value | 0.006 | <0.001 | <0.001 | 0.001 | <0.001 | <0.001 |
| Patterned area (μm^2^) |  | 8.8 | | | | | |
| Contact time (s) | | 5 | 20 | 60 | 120 | 240 | 360 |
| Median cell adhesion force (nN) | | 0.870 | 1.435 | 2.155 | 2.245 | 2.950 | 3.610 |
| Standard error (nN) | | 0.112 | 0.143 | 0.194 | 0.288 | 0.418 | 0.464 |
| Patterned area 5.3 μm^2^ | *P* value | 0.595 | 0.551 | 0.227 | 0.260 | 0.370 | 0.845 |
| Patterned area 2.4 μm^2^ | *P* value | 0.765 | 0.886 | 0.793 | 0.759 | 0.219 | 0.491 |
| Patterned area (μm^2^) |  | 5.3 | | | | | |
| Contact time (s) | | 5 | 20 | 60 | 120 | 240 | 360 |
| Median cell adhesion force (nN) | | 1.093 | 1.490 | 1.910 | 2.340 | 2.915 | 3.680 |
| Standard error (nN) | | 0.147 | 0.203 | 0.285 | 0.414 | 0.331 | 0.374 |
| Patterned area 2.4 μm^2^ | *P* value | 0.440 | 0.822 | 0.682 | 0.711 | 0.669 | 0.312 |
| Patterned area (μm^2^) |  | 2.4 | | | | | |
| Contact time (s) | | 5 | 20 | 60 | 120 | 240 | 360 |
| Median cell adhesion force (nN) | | 0.971 | 1.390 | 1.890 | 2.310 | 2.645 | 3.390 |
| Standard error (nN) | | 0.112 | 0.148 | 0.179 | 0.355 | 0.299 | 0.403 |

**Supplementary Table 4. Statistical analysis comparing the adhesion force of wt fibroblasts to printed fibronectin patterns with indicated substrate area at given contact time.** Given are the median and standard error of the adhesion force of wt fibroblasts to printed fibronectin patterns for different contact times. *P* values compare the adhesion force of wt fibroblasts to printed fibronectin pattern of given area with that measured of the indicated fibronectin pattern area at the given contact time. *P* values were calculated using two-sided Mann-Whitney tests, black values show significant difference (*P* < 0.05), and red values non-significant difference (*P* ≥ 0.05). Data taken from Fig. 3B.

| Comparison of the adhesion force of wt HeLa cells per substrate area | | | | | | | |
| --- | --- | --- | --- | --- | --- | --- | --- |
| Patterned area (μm^2^) | | 81.2 | | | | | |
| Contact time (s) | | 5 | 20 | 60 | 120 | 240 | 360 |
| Median cell adhesion force per area (nN μm^-2^) | | 0.007 | 0.009 | 0.013 | 0.018 | 0.020 | 0.026 |
| Standard error (nN μm^-2^) | | 0.002 | 0.001 | 0.003 | 0.002 | 0.004 | 0.006 |
| Patterned area 42.6 μm^2^ | *P* value | 0.007 | <0.001 | 0.005 | 0.012 | 0.005 | 0.035 |
| Patterned area 33.2 μm^2^ | *P* value | 0.011 | 0.004 | 0.006 | 0.097 | 0.157 | 0.325 |
| Patterned area 4.3 μm^2^ | *P* value | <0.001 | <0.001 | <0.001 | <0.001 | <0.001 | <0.001 |
| Patterned area 3.4 μm^2^ | *P* value | <0.001 | <0.001 | <0.001 | <0.001 | <0.001 | <0.001 |
| Patterned area (μm^2^) |  | 42.6 | | | | | |
| Contact time (s) | | 5 | 20 | 60 | 120 | 240 | 360 |
| Median cell adhesion force per area (nN μm^-2^) | | 0.012 | 0.017 | 0.020 | 0.026 | 0.034 | 0.036 |
| Standard error (nN μm^-2^) | | 0.001 | 0.001 | 0.002 | 0.003 | 0.004 | 0.005 |
| Patterned area 33.2 μm^2^ | *P* value | 0.952 | 0.850 | 0.754 | 0.342 | 0.320 | 0.147 |
| Patterned area 4.3 μm^2^ | *P* value | <0.001 | <0.001 | <0.001 | <0.001 | <0.001 | <0.001 |
| Patterned area 3.4 μm^2^ | *P* value | <0.001 | <0.001 | <0.001 | <0.001 | <0.001 | <0.001 |
| Patterned area (μm^2^) |  | 33.2 | | | | | |
| Contact time (s) | | 5 | 20 | 60 | 120 | 240 | 360 |
| Median cell adhesion force per area (nN μm^-2^) | | 0.014 | 0.021 | 0.028 | 0.031 | 0.034 | 0.044 |
| Standard error (nN μm^-2^) | | 0.002 | 0.002 | 0.002 | 0.003 | 0.006 | 0.007 |
| Patterned area 4.3 μm^2^ | *P* value | <0.001 | <0.001 | <0.001 | <0.001 | <0.001 | <0.001 |
| Patterned area 3.4 μm^2^ | *P* value | <0.001 | <0.001 | <0.001 | <0.001 | <0.001 | <0.001 |
| Patterned area (μm^2^) |  | 4.3 | | | | | |
| Contact time (s) | | 5 | 20 | 60 | 120 | 240 | 360 |
| Median cell adhesion force per area (nN μm^-2^) | | 0.134 | 0.142 | 0.208 | 0.261 | 0.275 | 0.352 |
| Standard error (nN μm^-2^) | | 0.009 | 0.017 | 0.020 | 0.023 | 0.043 | 0.034 |
| Patterned area 3.4 μm^2^ | *P* value | 0.028 | 0.005 | 0.342 | 0.06 | 0.072 | 0.010 |
| Patterned area (μm^2^) |  | 3.4 | | | | | |
| Contact time (s) | | 5 | 20 | 60 | 120 | 240 | 360 |
| Median cell adhesion force per area (nN μm^-2^) | | 0.191 | 0.222 | 0.333 | 0.400 | 0.544 | 0.600 |
| Standard error (nN μm^-2^) | | 0.010 | 0.014 | 0.016 | 0.024 | 0.055 | 0.035 |

**Supplementary Table 5. Statistical analysis comparing the adhesion force of wt HeLa cells per area of printed collagen I patterns with increasing contact time.** Given are the median and standard error of the adhesion force of wt HeLa cells per area of printed collagen I patterns. *P* values compare the adhesion force of wt HeLa cells to printed collagen I patterns of given areas with that measured of the indicated collagen I pattern area at the given contact time*. P* values were calculated using two-sided Mann-Whitney tests, black values show significant difference (*P* < 0.05), and red values non-significant difference (*P* ≥ 0.05)*.* Data taken from Fig. 4A.

| Comparison of the adhesion force of wt fibroblasts per substrate area | | | | | | | |
| --- | --- | --- | --- | --- | --- | --- | --- |
| Patterned area (μm^2^) | | 58.1 | | | | | |
| Contact time (s) | | 5 | 20 | 60 | 120 | 240 | 360 |
| Median cell adhesion force per area (nN μm^-2^) | | 0.020 | 0.042 | 0.058 | 0.068 | 0.086 | 0.094 |
| Standard error (nN μm^-2^) | | 0.003 | 0.005 | 0.006 | 0.008 | 0.012 | 0.015 |
| Patterned area 34.1 μm^2^ | *P* value | <0.001 | <0.001 | 0.001 | 0.001 | 0.003 | <0.001 |
| Patterned area 8.8 μm^2^ | *P* value | <0.001 | <0.001 | <0.001 | <0.001 | <0.001 | <0.001 |
| Patterned area 5.3 μm^2^ | *P* value | <0.001 | <0.001 | <0.001 | <0.001 | <0.001 | <0.001 |
| Patterned area 2.4 μm^2^ | *P* value | <0.001 | <0.001 | <0.001 | <0.001 | <0.001 | <0.001 |
| Patterned area (μm^2^) |  | 34.1 | | | | | |
| Contact time (s) | | 5 | 20 | 60 | 120 | 240 | 360 |
| Median cell adhesion force per area (nN μm^-2^) | | 0.040 | 0.066 | 0.107 | 0.117 | 0.149 | 0.170 |
| Standard error (nN μm^-2^) | | 0.005 | 0.006 | 0.010 | 0.011 | 0.017 | 0.015 |
| Patterned area 8.8 μm^2^ | *P* value | <0.001 | <0.001 | <0.001 | <0.001 | <0.001 | <0.001 |
| Patterned area 5.3 μm^2^ | *P* value | <0.001 | <0.001 | <0.001 | <0.001 | <0.001 | <0.001 |
| Patterned area 2.4 μm^2^ | *P* value | <0.001 | <0.001 | <0.001 | <0.001 | <0.001 | <0.001 |
| Patterned area (μm^2^) |  | 8.8 | | | | | |
| Contact time (s) | | 5 | 20 | 60 | 120 | 240 | 360 |
| Median cell adhesion force per area (nN μm^-2^) | | 0.099 | 0.163 | 0.245 | 0.255 | 0.335 | 0.410 |
| Standard error (nN μm^-2^) | | 0.013 | 0.016 | 0.022 | 0.033 | 0.048 | 0.053 |
| Patterned area 5.3 μm^2^ | *P* value | <0.001 | <0.001 | <0.001 | <0.001 | 0.007 | <0.001 |
| Patterned area 2.4 μm^2^ | *P* value | <0.001 | <0.001 | <0.001 | <0.001 | <0.001 | <0.001 |
| Patterned area (μm^2^) |  | 5.3 | | | | | |
| Contact time (s) | | 5 | 20 | 60 | 120 | 240 | 360 |
| Median cell adhesion force per area (nN μm^-2^) | | 0.228 | 0.302 | 0.364 | 0.470 | 0.496 | 0.694 |
| Standard error (nN μm^-2^) | | 0.028 | 0.038 | 0.087 | 0.050 | 0.062 | 0.071 |
| Patterned area 2.4 μm^2^ | *P* value | <0.001 | <0.001 | <0.001 | <0.001 | <0.001 | <0.001 |
| Patterned area (μm^2^) |  | 2.4 | | | | | |
| Contact time (s) | | 5 | 20 | 60 | 120 | 240 | 360 |
| Median cell adhesion force per area (nN μm^-2^) | | 0.404 | 0.579 | 0.788 | 0.963 | 1.215 | 1.413 |
| Standard error (nN μm^-2^) | | 0.047 | 0.062 | 0.075 | 0.148 | 0.125 | 0.168 |

**Supplementary Table 6. Statistical analysis comparing adhesion force of wt fibroblasts per area of printed fibronectin patterns with indicated surface areas at given contact times.** The median and standard error of the adhesion force of wt fibroblasts per area of printed fibronectin pattern. *P* values compare the adhesion force of wt fibroblasts to printed fibronectin pattern of given area with that measured of indicated fibronectin pattern area at the given contact time. *P* values were calculated by two-sided Mann-Whitney tests, black values show significant difference (*P* < 0.05)*.* Data taken from Fig. 4B.

| Comparison of the relative adhesion force of wt HeLa cells per substrate area | | | | | | | |
| --- | --- | --- | --- | --- | --- | --- | --- |
| Patterned area (μm^2^) | | 81.2 | | | | | |
| Contact time (s) | | 5 | 20 | 60 | 120 | 240 | 360 |
| Relative median cell adhesion force per area | | 0.701 | 0.802 | 0.790 | 0.861 | 0.752 | 0.684 |
| Standard error | | 0.182 | 0.109 | 0.164 | 0.108 | 0.162 | 0.165 |
| *P* value |  |  | 0.310 | 0.853 | 0.326 | 0.744 | 0.755 |
| Patterned area (μm^2^) | | 42.6 | | | | | |
| Contact time (s) | | 5 | 20 | 60 | 120 | 240 | 360 |
| Relative median cell adhesion force per area | | 1.298 | 1.463 | 1.222 | 1.103 | 0.939 | 0.845 |
| Standard error | | 0.111 | 0.104 | 0.107 | 0.153 | 0.179 | 0.132 |
| *P* value |  |  | 0.217 | 0.805 | 0.528 | 0.543 | 0.346 |
| Patterned area (μm^2^) | | 33.2 | | | | | |
| Contact time (s) | | 5 | 20 | 60 | 120 | 240 | 360 |
| Relative median of cell adhesion force per area | | 1.087 | 1.349 | 1.177 | 1.250 | 1.282 | 0.949 |
| Standard error | | 0.164 | 0.192 | 0.113 | 0.142 | 0.239 | 0.175 |
| *P* value |  |  | 0.464 | 0.912 | 0.914 | 0.510 | 0.115 |
| Patterned area (μm^2^) | | 4.3 | | | | | |
| Contact time (s) | | 5 | 20 | 60 | 120 | 240 | 360 |
| Relative median of cell adhesion force per area | | 8.406 | 7.273 | 7.511 | 7.482 | 6.260 | 5.496 |
| Standard error | | 0.976 | 1.439 | 1.185 | 1.106 | 1.635 | 0.881 |
| *P* value |  |  | 0.422 | 0.711 | 0.856 | 0.458 | 0.027 |
| Patterned area (μm^2^) | | 3.4 | | | | | |
| Contact time (s) | | 5 | 20 | 60 | 120 | 240 | 360 |
| Relative median of cell adhesion force per area | | 11.340 | 10.130 | 10.670 | 10.200 | 11.000 | 8.342 |
| Standard error | | 1.058 | 1.172 | 0.966 | 1.147 | 2.085 | 0.913 |
| *P* value |  |  | 0.759 | 0.280 | 0.609 | 0.768 | 0.006 |

**Supplementary Table 7. Statistical analysis comparing the adhesion force of wt HeLa cells per area of printed collagen I pattern and increasing contact time.** The median and standard error of the relative adhesion force of wt HeLa cells per area of printed collagen I patterns. The data was normalized to the mean cell adhesion force per area on the largest substrate pattern and contact time. *P* values compare the relative adhesion force of wt HeLa cells to printed collagen I patterns of given area at indicated contact time to that measured at 5 s contact time*.* *P* values were calculated using two-sided Mann-Whitney tests, black values show significant difference (*P* < 0.05), and red values non-significant difference (*P* ≥ 0.05). Data taken from Supplementary Fig. 3A.

| Comparison of the relative adhesion force of wt fibroblasts per substrate area | | | | | | |
| --- | --- | --- | --- | --- | --- | --- |
| Patterned area (μm^2^) | 58.1 | | | | | |
| Contact time (s) | 5 | 20 | 60 | 120 | 240 | 360 |
| Relative median cell adhesion force per area | 0.799 | 0.961 | 0.933 | 0.891 | 0.809 | 0.835 |
| Standard error | 0.104 | 0.105 | 0.096 | 0.109 | 0.115 | 0.129 |
| *P* value |  | 0.985 | 0.569 | 0.957 | 0.937 | 0.736 |
| Patterned area (μm^2^) | 34.1 | | | | | |
| Contact time (s) | 5 | 20 | 60 | 120 | 240 | 360 |
| Relative median cell adhesion force per area | 1.582 | 1.499 | 1.733 | 1.532 | 1.405 | 1.511 |
| Standard error | 0.198 | 0.129 | 0.160 | 0.143 | 0.156 | 0.129 |
| *P* value |  | 0.605 | 0.620 | 0.503 | 0.186 | 0.353 |
| Patterned area (μm^2^) | 8.8 | | | | | |
| Contact time (s) | 5 | 20 | 60 | 120 | 240 | 360 |
| Relative median cell adhesion force per area | 3.881 | 3.718 | 3.959 | 3.356 | 3.158 | 6.162 |
| Standard error | 0.501 | 0.371 | 0.357 | 0.430 | 0.448 | 0.468 |
| *P* value |  | 0.306 | 0.309 | 0.119 | 0.024 | 0.283 |
| Patterned area (μm^2^) | 5.3 | | | | | |
| Contact time (s) | 5 | 20 | 60 | 120 | 240 | 360 |
| Relative median cell adhesion force per area | 8.096 | 6.409 | 5.826 | 5.807 | 4.701 | 3.641 |
| Standard error | 1.087 | 0.874 | 1.406 | 0.652 | 0.589 | 0.626 |
| *P* value |  | 0.518 | 0.171 | 0.095 | 0.002 | 0.085 |
| Patterned area (μm^2^) | 2.4 | | | | | |
| Contact time (s) | 5 | 20 | 60 | 120 | 240 | 360 |
| Relative median cell adhesion force per area | 15.870 | 13.200 | 12.730 | 12.660 | 11.440 | 12.540 |
| Standard error | 1.839 | 1.402 | 1.205 | 1.948 | 1.175 | 1.492 |
| *P* value |  | 0.614 | 0.333 | 0.238 | 0.007 | 0.071 |

**Supplementary Table 8. Statistical analysis comparing the adhesion force of wt fibroblasts per area of printed** **fibronectin patterns at indicated contact time.** The median and standard error of the relative adhesion force of wt fibroblasts per area pf printed fibronectin pattern. The data was normalized to the mean cell adhesion force per area on the largest substrate pattern and contact time. *P* values compare the adhesion force of wt fibroblasts to printed fibronectin pattern of given area at indicated contact time to that measured at 5 s contact time. *P* values were calculated using two-sided Mann-Whitney tests, black values show significant difference (*P* < 0.05), and red values non-significant difference (*P* ≥ 0.05)*.* Data taken from Supplementary Fig. 3B.

| Comparison of the adhesion strengthening rate of wt HeLa cells to collagen I patterns | | | | | | |
| --- | --- | --- | --- | --- | --- | --- |
| Patterned area (μm^2^) | | 81.2 | 42.6 | 33.2 | 4.3 | 3.4 |
| Adhesion strengthening rate | | 0.0055 | 0.0040 | 0.0023 | 0.0019 | 0.0023 |
| Standard error | | 0.0008 | 0.0005 | 0.0004 | 0.0004 | 0.0003 |
| Patterned area 42.6 μm^2^ | *P* value | 0.122 |  |  |  |  |
| Patterned area 33.2 μm^2^ | *P* value | 0.003 | 0.010 |  |  |  |
| Patterned area 4.3 μm^2^ | *P* value | <0.001 | 0.001 | 0.476 |  |  |
| Patterned area 3.4 μm^2^ | *P* value | <0.001 | 0.004 | 0.946 | 0.462 |  |

**Supplementary Table 9. Statistical analysis comparing the adhesion strengthening rate of wt HeLa cells to collagen I patterns.** Adhesion strengthening rates of wt HeLa cells were quantified by the slope of a linear regression fit of the adhesion force to printed collagen I pattern for all contact times. *P* values compare the adhesion strengthening rates measured of wt HeLa cells to collagen I patterns having different areas. *P* values were calculated using one-sided extra sum-of-squares *F* test, black values show significant difference (*P* < 0.05) and red values non-significant difference (*P* ≥ 0.05)*.* Data taken from Fig. 4C.

| Comparison of the adhesion strengthening rate of wt fibroblasts to fibronectin patterns | | | | | | |
| --- | --- | --- | --- | --- | --- | --- |
| Patterned area (μm^2^) | | 58.1 | 34.1 | 8.8 | 5.3 | 2.4 |
| Adhesion strengthening rate | | 0.0134 | 0.0118 | 0.0081 | 0.0067 | 0.0062 |
| Standard error | | 0.0018 | 0.0013 | 0.0011 | 0.0011 | 0.0009 |
| Patterned area 34.1 μm^2^ | *P* value | 0.334 |  |  |  |  |
| Patterned area 8.8 μm^2^ | *P* value | 0.011 | 0.035 |  |  |  |
| Patterned area 5.3 μm^2^ | *P* value | 0.001 | 0.009 | 0.343 |  |  |
| Patterned area 2.4 μm^2^ | *P* value | <0.001 | 0.002 | 0.173 | 0.714 | 0.462 |

**Supplementary Table 10. Statistical analysis comparing the adhesion strengthening rate of wt fibroblasts to fibronectin patterns.** Adhesion strengthening rates of wt fibroblasts were quantified by the slope of a linear regression fit of wt fibroblasts adhesion force to printed fibronectin pattern for all contact times. *P* values compare the adhesion strengthening rates measured of wt fibroblasts to fibronectin patterns having different areas. *P* values were calculated using one-sided extra sum-of-squares *F* test, black values show significant difference (*P* < 0.05), and red values non-significant difference (*P* ≥ 0.05)*.* Data taken from Fig. 4C.

| Comparison of the adhesion force of TKO+THD HeLa cells to collagen I patterns | | | | | | | | |
| --- | --- | --- | --- | --- | --- | --- | --- | --- |
| Patterned area (μm^2^) | | | 81.2 | | | | | |
| Contact time (s) | | | 5 | 20 | 60 | 120 | 240 | 360 |
| Median cell adhesion force (nN) | | | 0.527 | 0.898 | 1.264 | 1.417 | 2.256 | 2.415 |
| Standard error (nN) | | | 0.054 | 0.084 | 0.099 | 0.151 | 0.125 | 0.221 |
| Patterned area 33.2 μm^2^ | | *P* value | 0.510 | 0.817 | 0.624 | 0.661 | 0.322 | 0.628 |
| Patterned area 3.4 μm^2^ | | *P* value | 0.331 | 0.561 | 0.541 | 0.945 | 0.145 | 0.568 |
| Patterned area (μm^2^) | | | 33.2 | | | | | |
| Contact time (s) | | | 5 | 20 | 60 | 120 | 240 | 360 |
| Median cell adhesion force (nN) | | | 0.545 | 0.842 | 1.009 | 1.388 | 1.883 | 2.553 |
| Standard error (nN) | | | 0.050 | 0.078 | 0.120 | 0.202 | 0.258 | 0.351 |
| Patterned area 3.4 μm^2^ | *P* value | | 0.142 | 0.442 | 0.326 | 0.772 | 0.531 | 0.580 |
| Patterned area (μm^2^) | | | 3.4 |  |  |  |  |  |
| Contact time (s) | | | 5 | 20 | 60 | 120 | 240 | 360 |
| Median cell adhesion force (nN) | | | 0.856 | 1.158 | 1.750 | 1.584 | 1.892 | 2.106 |
| Standard error (nN) | | | 0.093 | 0.106 | 0.172 | 0.234 | 0.262 | 0.265 |
| Comparison of the adhesion force of PXN KO HeLa cells to collagen I patterns | | | | | | | | |
| Patterned area (μm^2^) | | | 81.2 | | | | | |
| Contact time (s) | | | 5 | 20 | 60 | 120 | 240 | 360 |
| Median cell adhesion force (nN) | | | 0.755 | 1.096 | 1.539 | 2.132 | 2.673 | 3.698 |
| Standard error (nN) | | | 0.084 | 0.128 | 0.329 | 0.585 | 0.869 | 0.815 |
| Patterned area 33.2 μm^2^ | | *P* value | 0.033 | 0.039 | 0.048 | 0.027 | 0.048 | 0.017 |
| Patterned area 3.4 μm^2^ | | *P* value | 0.021 | 0.257 | 0.089 | 0.008 | 0.017 | 0.037 |
| Patterned area (μm^2^) | | | 33.2 | | | | | |
| Contact time (s) | | | 5 | 20 | 60 | 120 | 240 | 360 |
| Median cell adhesion force (nN) | | | 0.429 | 0.651 | 0.839 | 1.025 | 1.653 | 1.935 |
| Standard error (nN) | | | 0.084 | 0.209 | 0.389 | 0.408 | 0.569 | 0.327 |
| Patterned area 3.4 μm^2^ | *P* value | | 0.660 | 0.116 | 0.464 | 0.708 | 0.721 | 0.458 |
| Patterned area (μm^2^) | | | 3.4 |  |  |  |  |  |
| Contact time (s) | | | 5 | 20 | 60 | 120 | 240 | 360 |
| Median cell adhesion force (nN) | | | 0.517 | 0.921 | 1.020 | 1.227 | 1.542 | 2.250 |
| Standard error (nN) | | | 0.043 | 0.085 | 0.121 | 0.130 | 0.242 | 0.413 |

**Supplementary Table 11. Statistical analysis comparing the adhesion force of TKO+THD and PXN KO HeLa cells to indicated collagen I patterns at given contact time.** Given are the median and standard error of the adhesion forces of talin1/2-depleted expressing talin1 head domain (TKO+THD) and paxillin-depleted (PXN KO) HeLa cells to indicated collagen I patterns at different contact times. *P* values comparing adhesion forces of TKO+THD or PXN KO HeLa cells to collagen I pattern with indicated area at the given contact time. *P* values were calculated using two-sided Mann-Whitney tests, black values show significant difference (*P* < 0.05), and red values non-significant difference (*P* ≥ 0.05). Data taken from Supplementary Fig. 13A and C.

| Comparison of the adhesion force of TKO+THD fibroblasts to fibronectin patterns | | | | | | | | |
| --- | --- | --- | --- | --- | --- | --- | --- | --- |
| Patterned area (μm^2^) | | | 58.1 | | | | | |
| Contact time (s) | | | 5 | 20 | 60 | 120 | 240 | 360 |
| Median cell adhesion force (nN) | | | 0.750 | 0.847 | 2.098 | 2.637 | 4.072 | 4.478 |
| Standard error (nN) | | | 0.085 | 0.147 | 0.296 | 0.289 | 0.461 | 0.571 |
| Patterned area 8.8 μm^2^ | | *P* value | 0.209 | 0.215 | 0.411 | 0.318 | 0.334 | 0.431 |
| Patterned area 2.4 μm^2^ | | *P* value | 0.078 | 0.304 | 0.002 | 0.004 | 0.004 | 0.009 |
| Patterned area (μm^2^) | | | 8.8 | | | | | |
| Contact time (s) | | | 5 | 20 | 60 | 120 | 240 | 360 |
| Median cell adhesion force (nN) | | | 0.981 | 1.443 | 1.590 | 1.981 | 2.958 | 4.078 |
| Standard error (nN) | | | 0.151 | 0.298 | 0.380 | 0.493 | 0.573 | 0.603 |
| Patterned area 2.4 μm^2^ | *P* value | | 0.005 | 0.008 | 0.022 | 0.025 | 0.036 | 0.172 |
| Patterned area (μm^2^) | | | 2.4 |  |  |  |  |  |
| Contact time (s) | | | 5 | 20 | 60 | 120 | 240 | 360 |
| Median cell adhesion force (nN) | | | 0.589 | 0.986 | 1.123 | 1.453 | 1.960 | 2.457 |
| Standard error (nN) | | | 0.080 | 0.115 | 0.127 | 0.178 | 0.262 | 0.553 |
| Comparison of the adhesion force of PXN KO fibroblasts to fibronectin patterns | | | | | | | | |
| Patterned area (μm^2^) | | | 58.1 | | | | | |
| Contact time (s) | | | 5 | 20 | 60 | 120 | 240 | 360 |
| Median cell adhesion force (nN) | | | 1.004 | 1.498 | 2.553 | 3.232 | 4.717 | 6.063 |
| Standard error (nN) | | | 0.157 | 0.237 | 0.373 | 0.567 | 1.308 | 1.292 |
| Patterned area 8.8 μm^2^ | | *P* value | 0.045 | 0.300 | 0.017 | 0.086 | 0.022 | 0.036 |
| Patterned area 2.4 μm^2^ | | *P* value | 0.806 | 0.713 | 0.539 | 0.038 | 0.026 | 0.008 |
| Patterned area (μm^2^) | | | 8.8 | | | | | |
| Contact time (s) | | | 5 | 20 | 60 | 120 | 240 | 360 |
| Median cell adhesion force (nN) | | | 0.788 | 1.310 | 1.973 | 2.646 | 3.683 | 4.110 |
| Standard error (nN) | | | 0.066 | 0.190 | 0.216 | 0.404 | 0.448 | 0.677 |
| Patterned area 2.4 μm^2^ | *P* value | | 0.290 | 0.738 | 0.331 | 0.891 | 0.896 | 0.983 |
| Patterned area (μm^2^) | | | 2.4 |  |  |  |  |  |
| Contact time (s) | | | 5 | 20 | 60 | 120 | 240 | 360 |
| Median cell adhesion force (nN) | | | 0.956 | 1.512 | 2.730 | 3.131 | 3.768 | 4.320 |
| Standard error (nN) | | | 0.195 | 0.337 | 0.446 | 0.283 | 0.460 | 0.648 |

**Supplementary Table 12. Statistical analysis comparing the adhesion force of TKO+THD and PXN KO fibroblasts to indicated fibronectin patterns at given contact time.** Given are the median and standard error of the adhesion forces of talin1/2-depleted expressing talin1 head domain (TKO+THD) and paxillin-depleted (PXN KO) fibroblasts to indicated fibronectin patterns at different contact times. *P* values comparing adhesion forces of TKO+THD or PXN KO fibroblasts to fibronectin pattern with indicated area at the given contact time. *P* values were calculated using two-sided Mann-Whitney tests, black values show significant difference (*P* < 0.05), and red values non-significant difference (*P* ≥ 0.05). Data taken from Supplementary Fig. 13B and D.

**References**

1. Krieg, M., Helenius, J., Heisenberg, C. & Muller, D. J. A Bond for a Lifetime: Employing Membrane Nanotubes from Living Cells to Determine Receptor–Ligand Kinetics. *Angew. Chem.* **120**, 9921–9923 (2008).
