# Supplementary material for: Mammalian cells measure the extracellular matrix area and respond through switching the adhesion state
Source: Nat Commun. 2025 Jul 25;16:6870. doi: 10.1038/s41467-025-62153-7 (PMC12297719; doi:10.1038/s41467-025-62153-7)
Supplement: Supplementary file 2 — Reporting Summary [file 41467_2025_62153_MOESM2_ESM.pdf]

nature portfolio

Corresponding author(s): Nico Strohmeyer, Daniel J. Müller

Last updated by author(s): Jun 24, 2025

Reporting Summary

Nature Portfolio wishes to improve the reproducibility of the work that we publish. This form provides structure for consistency and transparency in reporting. For further information on Nature Portfolio policies, see our Editorial Policies and the Editorial Policy Checklist.

Statistics

For all statistical analyses, confirm that the following items are present in the figure legend, table legend, main text, or Methods section.

n/a	Confirmed	
☐	☒	The exact sample size (n) for each experimental group/condition, given as a discrete number and unit of measurement
☐	☒	A statement on whether measurements were taken from distinct samples or whether the same sample was measured repeatedly
☐	☒	The statistical test(s) used AND whether they are one- or two-sided<br>*Only common tests should be described solely by name; describe more complex techniques in the Methods section.*
☒	☐	A description of all covariates tested
☒	☐	A description of any assumptions or corrections, such as tests of normality and adjustment for multiple comparisons
☐	☒	A full description of the statistical parameters including central tendency (e.g. means) or other basic estimates (e.g. regression coefficient) AND variation (e.g. standard deviation) or associated estimates of uncertainty (e.g. confidence intervals)
☐	☒	For null hypothesis testing, the test statistic (e.g. F, t, r) with confidence intervals, effect sizes, degrees of freedom and P value noted<br>*Give P values as exact values whenever suitable.*
☒	☐	For Bayesian analysis, information on the choice of priors and Markov chain Monte Carlo settings
☒	☐	For hierarchical and complex designs, identification of the appropriate level for tests and full reporting of outcomes
☒	☐	Estimates of effect sizes (e.g. Cohen's d, Pearson's r), indicating how they were calculated

Our web collection on statistics for biologists contains articles on many of the points above.

Software and code

Policy information about availability of computer code

Data collection	For SCFS experiments, a JPK inbuilt data acquisition software was used (Versions 4.3.55 and 5.0135). For Flow Cytometry, a BD Bioscience inbuilt software (BD FACSDiva 8.1) was used. For confocal laser scanning microscopy Zeiss (ZenBlue, version 3.5.093.00008) software was used.
Data analysis	Statistical tests were performed using Prism (GraphPad Software - Version 8.4.3 (471)). Adhesion forces and single molecule interactions used for adhesion probabilities were determined using the JPK data analysis software (JPK Instruments, version spm-4.3.55). Adhesion force strengthening was determined using PRISM (Version 8.4.3 (471)). Fluorescence intensities for flow cytometry experiments were determined using the FLowJo (FlowJo10). Images for confocal imaging were processed using ZEN Blue software (Zeiss, version 3.5.093.00008)

For manuscripts utilizing custom algorithms or software that are central to the research but not yet described in published literature, software must be made available to editors and reviewers. We strongly encourage code deposition in a community repository (e.g. GitHub). See the Nature Portfolio guidelines for submitting code & software for further information.

Data

Policy information about availability of data

All manuscripts must include a data availability statement. This statement should provide the following information, where applicable:

- Accession codes, unique identifiers, or web links for publicly available datasets
- A description of any restrictions on data availability
- For clinical datasets or third party data, please ensure that the statement adheres to our policy

Source data are provided with this paper. The data generated in this study have been deposited in the ETH research collection available under

Research involving human participants, their data, or biological material

Policy information about studies with human participants or human data. See also policy information about sex, gender (identity/presentation), and sexual orientation and race, ethnicity and racism.

Reporting on sex and gender	N/A
Reporting on race, ethnicity, or other socially relevant groupings	N/A
Population characteristics	N/A
Recruitment	N/A
Ethics oversight	N/A

Note that full information on the approval of the study protocol must also be provided in the manuscript.

Field-specific reporting

Please select the one below that is the best fit for your research. If you are not sure, read the appropriate sections before making your selection.

- [x] Life sciences
- [ ] Behavioural & social sciences
- [ ] Ecological, evolutionary & environmental sciences

For a reference copy of the document with all sections, see nature.com/documents/nr-reporting-summary-flat.pdf

Life sciences study design

All studies must disclose on these points even when the disclosure is negative.

Sample size	Sample size was not predetermined. The adhesion of at least 15 cells was measured per condition to obtain statistically firm results. We estimated that this number of cells would suffice for biologically relevant differences. For fluorescence intensities, 20'000 cells per sample and a minimum of 3 samples was analyzed to attain statistically firm results. We estimated that this number of cells would suffice for biologically relevant differences. Confocal imaging was performed on at least 10 samples to verify the appearance of any relevant phenotypes.
Data exclusions	Data was not excluded from the analyses except in the case of technical issues during experimentation resulting in low quality results not meeting quality criteria.
Replication	Adhesion forces of at least 15 cells per condition and contact time were measured on at least three different days. For confocal imaging at least 10 cells in at least three different samples were analyzed . Flow cytometry experiments were performed on at least three samples with each containing 20'000 cells on at least three different days.
Randomization	The order of contact times was randomized for every cell and condition. Samples used for flow cytometry were analyzed in a random order and in alternation with other samples tested during the same experiment. Samples were allocated randomly into experimental groups on any given day
Blinding	Blinding was not relevant to this study since measurements (cell adhesion/flow cytometry/microscopy) were analyzed after data acquisition and were therefor not susceptible to experimenter bias. The same person collected and analyzed the data.

Reporting for specific materials, systems and methods

We require information from authors about some types of materials, experimental systems and methods used in many studies. Here, indicate whether each material, system or method listed is relevant to your study. If you are not sure if a list item applies to your research, read the appropriate section before selecting a response.

Materials & experimental systems

n/a	Involved in the study	
☐	☒	Antibodies
☐	☒	Eukaryotic cell lines
☒	☐	Palaeontology and archaeology
☒	☐	Animals and other organisms
☒	☐	Clinical data
☒	☐	Dual use research of concern
☒	☐	Plants

Methods

n/a	Involved in the study	
☒	☐	ChIP-seq
☐	☒	Flow cytometry
☒	☐	MRI-based neuroimaging

Antibodies

Antibodies used	Anti-Paxillin antibody [Y113], abcam, ab32084, western blot (1:1000), immunofluorescence (1:100), lot.no. 1085465-8;<br>Talin antibody TA205, bio-rad, MCA725G, western blot (1:1000), lot.no. 161369;<br>Rhodamine Phalloidin, thermofisher, R415, immunofluorescence (1:1000), 2892779;<br>Anti-GAPDH, abcam, ab9485, western blot (1:2500), lot.no. 1064471-1;<br>Goat anti-rabbit HRP, abcam, ab205718, western blot (1:2500), lot.no. 1059359-1;<br>Goat anti-mouse HRP, abcam, ab205719, western blot (1:2500), lot.no. 1093066-3;<br>FITC anti-Integrin alpha 1 [TS2/7], abcam, ab34176, flow cytometry (1:100), lot.no. 1059201-1;<br>FITC anti-human CD49b Antibody, biolegend, 359305, flow cytometry (1:200), lot.no. B360302 359305;<br>FITC anti-Integrin beta 1, thermofisher, 11-0291-82, flow cytometry (1:40), lot.no. 2702188;<br>anti-Integrin alpha 5, abcam, ab25189, flow cytometry (1:100), lot.no. R3222343-1;<br>Streptavidin-Phycoerythrin (R-PE), abcam, ab239759, flow cytometry (1:1000), lot.no. 1037919-3;<br>anti-Integrin alpha V, thermofisher, 12-0512-82, flow cytometry (1:100), lot.no. 2925558.<br>Donkey anti rabbit Alexa Fluor 488, abcam,ab150073, immunofluorescence (1:200), lot.no. 1005425-58<br>Monoclonal anti-talin antibody produced in mouse, Sigma, T3287, western blot (1:100), lot.not.0000174814;<br>anti-talin1 antibody [97H6], abcam, ab108480, western blot (1:500), lot.no.1053247-14;<br>anti-talin 2 antibody [68E7], abcam, ab105458, western blot (1:500), lot.no. 1034293-1.
Validation	All the commercially available antibodies used in this study were validated by the manufacturers and or previous publications through western blot flow cytometry or immunofluorescence.<br>Anti-Paxillin antibody [Y113], abcam, ab32084, https://www.abcam.com/en-us/products/primary-antibodies/paxillin-antibody-y113-ab32084;<br>Talin antibody | TA205, bio-rad, MCA725G, https://www.bio-rad-antibodies.com/monoclonal/human-talin-antibody-ta205-mca725.html?evCntryLang=CH-en&JSESSIONID_STERLING=adummyvalue;<br>Rhodamine Phalloidin, thermofisher, R415, https://www.thermofisher.com/order/catalog/product/R415;<br>Anti-GAPDH, abcam, ab9485, https://www.abcam.com/en-us/products/primary-antibodies/gapdh-antibody-loading-control-ab9485;<br>FITC anti-Integrin alpha 1 [TS2/7], abcam, ab34176, https://www.abcam.com/en-us/products/primary-antibodies/fitc-integrin-alpha-1-antibody-ts2-7-ab34176;<br>FITC anti-human CD49b Antibody, biolegend, 359305, https://www.biolegend.com/en-us/products/fitc-anti-human-cd49b-antibody-8880;<br>FITC anti-Integrin beta 1, thermofisher, 11-0291-82, https://www.thermofisher.com/antibody/product/CD29-Integrin-beta-1-Antibody-clone-eBioHMb1-1-HMb1-1-Monoclonal/11-0291-82;<br>anti-Integrin alpha 5, abcam, ab25189, https://www.abcam.com/en-us/products/primary-antibodies/biotin-integrin-alpha-5-antibody-5h10-27-ab25189.

Eukaryotic cell lines

Policy information about cell lines and Sex and Gender in Research

Cell line source(s)	Wild type HeLa (Kyoto) (kind gift from A. Hyman, MPI Molecular Cell Biology and Genetics, Germany), TKO HeLa cells, TKO + THD HeLa cells, KKO HeLa cells, PXN KO HeLa cells, wild type mouse embryonic kidney fibroblasts, TKO fibroblasts, TKO + THD fibroblasts (kind gift from C. Grashoff, University of Munster, Germany), KKO fibroblasts and PXN KO fibroblasts were used.
Authentication	Parental cell lines were not authenticated and engineered cell lines were authenticated by flow cytometry or western blot.
Mycoplasma contamination	Cells were tested negative for mycoplasma contamination.
Commonly misidentified lines<br>(See ICLAC register)	No cell line used in the paper is listed in the ICLAC database

Plants

Seed stocks	N/A
Novel plant genotypes	N/A
Authentication	N/A

Flow Cytometry

Plots

Confirm that:

- [x] The axis labels state the marker and fluorochrome used (e.g. CD4-FITC).
- [x] The axis scales are clearly visible. Include numbers along axes only for bottom left plot of group (a 'group' is an analysis of identical markers).
- [x] All plots are contour plots with outliers or pseudocolor plots.
- [x] A numerical value for number of cells or percentage (with statistics) is provided.

Methodology

Sample preparation	Cells were serum starved for at least 1 h, trypsinized, washed with PBS and 6 x 105 cells were resuspended in 500 µl PBS. Unlabeled wt HeLa cells and fibroblasts were analyzed as a negative control. TKO, KKO, TKO + THD, PXN KO or wt HeLa cells were incubated with antibody against integrin α1 (1:100; FITC anti-integrin α1, ab34176, Abcam), α2 (1:200; FITC anti-human CD49b, 359305, Biolegend), β1 (1:40; anti-integrin β1, 11-0291-82, Thermo Fisher Scientific) in PBS on ice for 1h. TKO, KKO, TKO + THD, PXN KO or wt fibroblasts were incubated with antibody against integrin α5 (1:100; anti-integrin α5, ab25189, Abcam), αV (1:100; anti-integrin αV, 12-0512-82, Thermo Fisher Scientific), β1 (1:40; anti-integrin β1, 11-0291-82, Thermo Fisher Scientific) in PBS on ice for 30 min. Cells incubated with antibodies against integrin α5 were washed with PBS twice and incubated with streptavidin-phycoerythrin (1:1000; ab239759, Abcam) in PBS on ice for 30 min. Following antibody incubation cells were washed twice with cold PBS and finally resuspended in 250 µl PBS and kept on ice. Fluorescence intensities of single cells were analyzed using a flow cytometer (Fortessa, BD Bioscience). Laser intensities were optimized for each experiment and maintained constant for conditions to be compared.
Instrument	Flow cytometry was carried out using the BD LSR Fortessa SORP or BD FACSMelody (BD Biosciences, USA).
Software	FlowJo V10 was used to analyze flow cytometry data.
Cell population abundance	After excluding debris and doublets 100% of cells were relevant for the study, as we used cell culture condition (100% purity of cells of interest).
Gating strategy	Flow cytometry data was gated according to forward and side scatter to exclude debris and doublets.

- [x] Tick this box to confirm that a figure exemplifying the gating strategy is provided in the Supplementary Information.
